# Type I interferon signaling attenuates regulatory T cell function in viral infection and in the tumor microenvironment

**DOI:** 10.1371/journal.ppat.1006985

**Published:** 2018-04-19

**Authors:** Arunakumar Gangaplara, Craig Martens, Eric Dahlstrom, Amina Metidji, Ameya S. Gokhale, Deborah D. Glass, Maria Lopez-Ocasio, Rachel Baur, Kishore Kanakabandi, Stephen F. Porcella, Ethan M. Shevach

**Affiliations:** 1 Laboratory of Immunology, National Institute of Allergy and Infectious Diseases, National Institutes of Health, Bethesda, MD, United States of America; 2 Genomics Unit, Research Technologies Section, Rocky Mountain Laboratories, National Institute of Allergy and Infectious Diseases, National Institutes of Health, Hamilton, MT, United States of America; 3 The Francis Crick Institute, London, United Kingdom; St. Jude Children's Research Hospital, UNITED STATES

## Abstract

Regulatory T cells (Tregs) play a cardinal role in the immune system by suppressing detrimental autoimmune responses, but their role in acute, chronic infectious diseases and tumor microenvironment remains unclear. We recently demonstrated that IFN-α/β receptor (IFNAR) signaling promotes Treg function in autoimmunity. Here we dissected the functional role of IFNAR-signaling in Tregs using Treg-specific IFNAR deficient (IFNAR^fl/fl^xFoxp3^YFP-Cre^) mice in acute LCMV Armstrong, chronic Clone-13 viral infection, and in tumor models. In both viral infection and tumor models, IFNAR^fl/fl^xFoxp3^YFP-Cre^ mice Tregs expressed enhanced Treg associated activation antigens. LCMV-specific CD8^+^ T cells and tumor infiltrating lymphocytes from IFNAR^fl/fl^xFoxp3^YFP-Cre^ mice produced less antiviral and antitumor IFN-γ and TNF-α. In chronic viral model, the numbers of antiviral effector and memory CD8^+^ T cells were decreased in IFNAR^fl/fl^xFoxp3^YFP-Cre^ mice and the effector CD4^+^ and CD8^+^ T cells exhibited a phenotype compatible with enhanced exhaustion. IFNAR^fl/fl^xFoxp3^YFP-Cre^ mice cleared Armstrong infection normally, but had higher viral titers in sera, kidneys and lungs during chronic infection, and higher tumor burden than the WT controls. The enhanced activated phenotype was evident through transcriptome analysis of IFNAR^fl/fl^xFoxp3^YFP-Cre^ mice Tregs during infection demonstrated differential expression of a unique gene signature characterized by elevated levels of genes involved in suppression and decreased levels of genes mediating apoptosis. Thus, IFN signaling in Tregs is beneficial to host resulting in a more effective antiviral response and augmented antitumor immunity.

## Author summary

Type I interferons (IFNs) play a predominant role in the immune response to infectious pathogens. The cellular targets of IFNs have been difficult to dissect because of the ubiquitous expression of the type I interferon receptor (IFNAR). The immune response of mice to lymphocytic choriomeningitis virus (LCMV) is one of the major models for analyzing the action of IFNs. Regulatory T cells (Tregs) have been implicated in the control of LCMV and it has been proposed that IFN may influence their function. The major goal of this study was to define the contribution of IFN signaling on Treg function during different stages LCMV infection. Tregs from mice with selective deletion of IFNAR manifested enhanced suppressive activity during acute/chronic LCMV infection resulting in increased CD8 T cell anergy, defective generation of memory T cells and persistence of virus. Similar effects of IFNAR signaling in Tregs were seen in a tumor model. We identified a unique set of genes in Tregs modulated by IFN signaling that may contribute to the enhanced suppressive function of IFNAR deficient Tregs. IFNs play a beneficial role during acute/chronic viral infections not only by contributing to viral clearance but also by attenuating the function of Tregs.

## Introduction

Regulatory T cells (Tregs) are a subset of CD4^+^ T cells, which express the transcription factor Foxp3, and are critical in forestalling both self- and non-self-reactive immune responses [1, 2]. Tregs primarily mediate their suppressive function by targeting conventional effector T cell activation and differentiation, mainly by decreasing the functional activity of antigen presenting cells (APCs) [3]. The critical role of Tregs in autoimmunity is best observed in scurfy mice or patients with IPEX syndrome that are completely deficient in Tregs and succumb to systemic autoimmune disease at a young age. While Tregs must control the activation of T effector cells to prevent autoimmunity, it is also clear that enhanced activation of Tregs may result in the inhibition of host immunity directed against microbes (virus, bacteria, protozoa, fungi and helminth) or tumors leading to poor antimicrobial or antitumor immune response with the persistence of pathogens, and defective tumor immunity [4–6].

Many animal models of bacterial infection are characterized by the expansion of Foxp3^+^ Tregs including *Listeria monocytogenes*, *Salmonella enterica*, and *Mycobacterium tuberculosis* infections and the suppressive function of Tregs can result in increased bacterial load with systemic tissue invasion [7–9]. Similarly in viral infection, higher frequencies of Tregs are associated with enhanced titers of Hepatitis C virus RNA and Dengue virus [10, 11]. Paradoxically, Tregs have been described to play an early protective role in local infection in animals models of Herpes simplex virus 2 and West Nile virus [12, 13]. During early phases of human immunodeficiency virus infection, Tregs have been postulated to control virus replication in target CD4^+^ T cells [14]. On the other hand Tregs may play an important beneficial role in preventing exuberant inflammatory responses during infection with parasites such as *Pneumocystis carinii* [15] and *Schistosoma mansoni* [16]. Similarly, Tregs protect the host from parasitic infections such as *Plasmodium* sp., *Toxoplasma gondii*, as well as infection with the fungus, *Candida albicans* [17–19]. These complex roles played by Tregs during acute and chronic microbial infections necessitate a delicate balance between the Foxp3^+^ Tregs and effector T cells to mount effective immune responses against pathogens without the induction of destructive autoimmunity.

The immune response towards viruses and intracellular bacteria are mediated by type I interferons (IFNs) which control the replication of pathogens within host cells. IFNs are members of a multi-gene family of cytokines, which encode IFN-α and IFN-β. Both IFN-α and IFN-β signal through a shared common heterodimeric receptor IFN-α/β receptor (IFNAR) composed of IFNAR1 and IFNAR2 [20]. The interactions of IFNs with the IFNAR mediates activation of Janus family protein kinases to induce the phosphorylation of signal transducer and activator of transcription (STAT). The canonical pathway of Type I IFN signaling is initiated by phosphorylation of STATs (STAT1, STAT2), induction of IFN-regulatory factor-9, resulting in the formation of a tri-molecular complex, IFN-stimulated gene factor-3, which translocates into the nucleus to induce transcription of IFN-stimulated genes through binding of IFN-stimulated response elements [21]. Additionally IFNAR signaling can trigger non-canonical pathways such as activation of γ-activated sequences through homodimerization of STATs (STAT1, STAT3, STAT4, STAT5, STAT6), phosphoinositide-3-kinase/mammalian target of rapamycin pathway, and mitogen-activated protein kinase pathway [22].

IFNs may mediate an array of host protective functions including restricting viral replication [23], activation of NK cell cytotoxicity, maturation of APCs, clonal expansion and survival of antigen-specific CD4 and CD8 T cells during viral infection, promotion of B cell responses, and induction of apoptosis [24–30]. Type I IFNs have proven to be clinically useful in the treatment of chronic viral infections and certain types of leukemias [31]. Detrimental effects of type I IFNs have also been extensively documented during viral infections as well as during bacterial, fungal and parasitic infections [32]. One of the best examples of the complex regulation of antiviral immunity by type I IFNs is lymphocytic choriomeningitis virus infection (LCMV). Blockade of IFN signaling in acute infection with LCMV Armstrong infection results in abrogation of CD8^+^ T cell responses and defective control of infection [33]. In contrast, blockade of IFN signaling during persistent LCMV Clone (Cl)-13 infection diminished immunosuppressive signals and decreased levels of IL-10 and PD-L1 expressing immunoregulatory DCs. Virus titers in both serum and kidneys were also reduced. The cell type (s) mediating the immunosuppressive effects of IFN have not been defined [33, 34].

Studies on the effects of type I IFNs on Treg function yielded conflicting results [35, 36] and have not used experimental systems to examine the direct effects of IFNs on Treg cell homeostasis and functions. Recently, Srivastava et al (2014) demonstrated that mice infected with LCMV Armstrong manifested a decrease in the absolute numbers of splenic Tregs between days 4 and 7 post infection and that this reduction correlated with the expansion of both CD4^+^ and CD8^+^ T effector cells which peak on day 7 post infection. Furthermore, they also demonstrated a selective depletion of wild type (WT) Tregs on day 7 post infection of mixed bone marrow chimeras between WT mice and mice with a global deletion of IFNAR (IFNAR^-/-^). This latter result is difficult to interpret as our recent studies [37] have shown that IFNAR^-/-^ Tregs in such chimeric (IFNAR^-/-^ x WT) mice are at a competitive disadvantage as are IFNAR^-/-^ Tregs in heterozygous female IFNAR^fl/fl^ x Foxp3^Cre/WT^ mice.

In this study, we used IFNAR^fl/fl^ x Foxp3^YFP-Cre^ mice to determine the role of IFNAR signaling specifically in Tregs during acute and chronic LCMV infection as well as in models of colon adenocarcinoma and melanoma. We demonstrate that IFNAR signaling in Tregs during the course of both acute and chronic viral infection results in a decrease in their activation status and a decrease in their suppressive function *in vivo*. The hypersuppressive state of Tregs in the absence of IFNAR signaling results in decreased CD8^+^ effector T cells, enhanced T effector cell exhaustion, defective generation of antiviral memory CD8^+^ T cells, and enhanced LCMV persistence. Similarly, in the tumor models, enhanced tumor growth and failure to efficiently generate antitumor T effector cells were observed in the absence of IFNAR signaling in Tregs. The enhanced suppressor function in the absence of IFNAR signaling in Tregs was accompanied by the induction of a gene expression pattern which was similar in the acute and chronic infection models and may be responsible for the heightened suppressor function.

## Results

### IFNAR signaling in Tregs modulates the generation of antiviral effector T cells in acute LCMV infection

**Fig 1:**
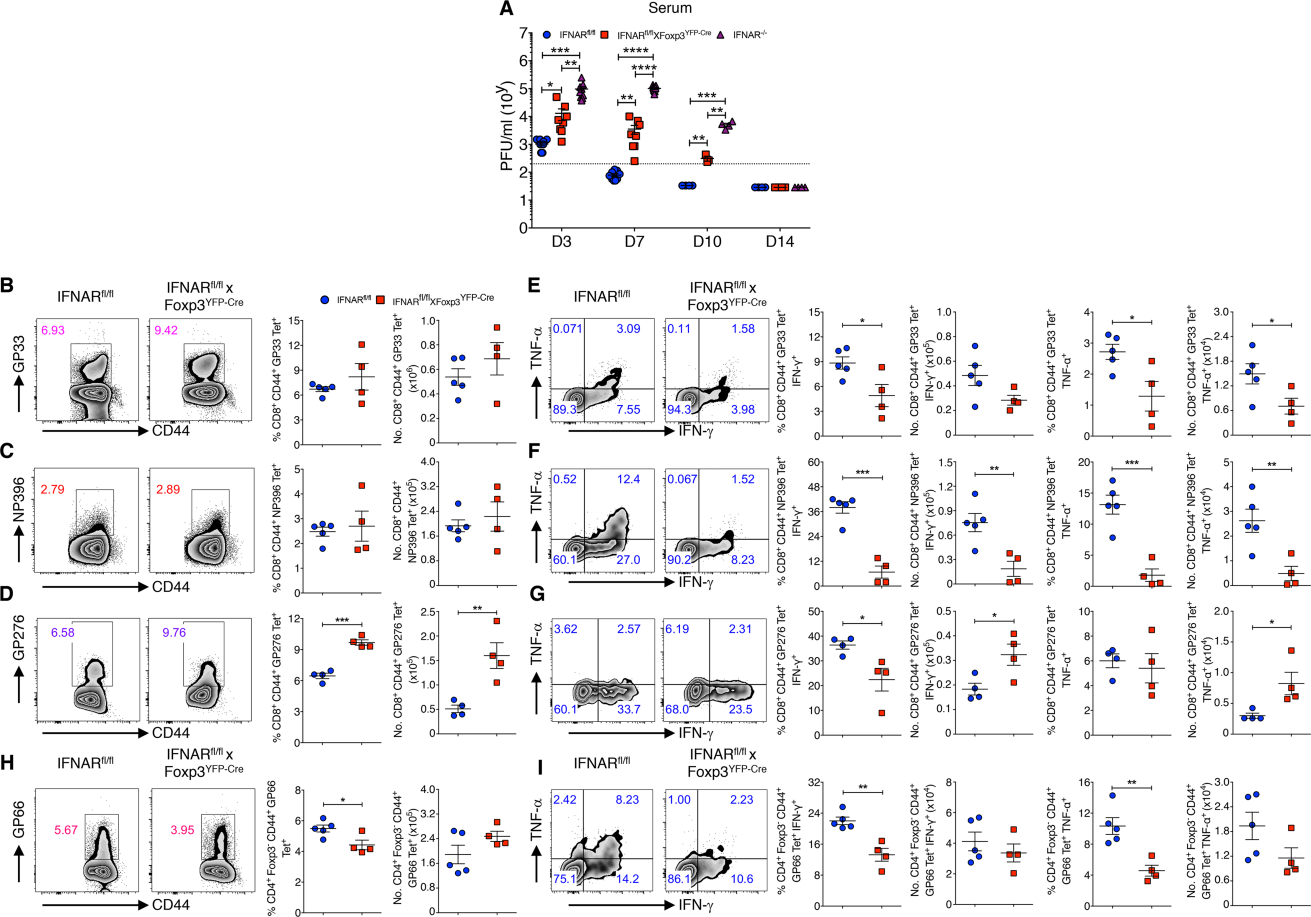
IFNAR signaling in Tregs modulates the generation of antiviral effector T cells in acute LCMV infection. (**A**) LCMV titers were determined from Armstrong virus infected IFNAR^fl/fl^, IFNAR^fl/fl^ x Foxp3^YFP-Cre^ and IFNAR^-/-^ mice sera on indicated days. (**B-D**) Splenocytes harvested from day 14 virus infected IFNAR^fl/fl^ and IFNAR^fl/fl^ x Foxp3^YFP-Cre^ mice were stained for GP33, NP396 and GP276 tetramers and analyzed for Tet^+^ T cells within CD8^+^CD44^+^ cells. (**E-G**) Spleen cells stimulated with GP33, NP396, GP276 and Golgi Stop for 5 hours at 37 ^o^C. Frequencies and absolute numbers of IFN-γ^+^ and TNF-α^+^ cytokine producing cells within CD8^+^CD44^+^GP33 Tet^+^, CD8^+^CD44^+^NP396 Tet^+^ and CD8^+^CD44^+^GP276 Tet^+^ T cells from day 14 acute LCMV infected mice are shown. (**H** and **I**) Spleen cells were analyzed (as in **B**-**D**) for GP66 Tet^+^ T cells, and IFN-γ^+^ and TNF-α^+^ producing cells were assessed among gated CD4^+^Foxp3^-^CD44^+^GP66 Tet^+^ T cells from day 14 acute LCMV infected mice. * *P* < 0.05, ** *P* < 0.01, *** *P* < 0.001, and **** *P* < 0.0001 (unpaired two-tailed Student’s *t*-test). Data are shown from two to three experiments involving three to nine mice per group (**A**), and representative of two independent experiments (**B-I)** with four to five mice per group (Mean±SEM).

Studies performed by Srivastava et al. (2014), provided preliminary evidence that IFNAR signaling inhibits the function of Tregs during an acute LCMV infection and that the absence of this inhibitory effect resulted in enhanced Treg function and impaired antiviral effector T cell function. Because this study did not definitively prove that the target of the suppressive function of IFNAR signaling was the Foxp3^+^ Treg, we generated Treg-lineage specific IFNAR deficient mice by crossing IFNAR^fl/fl^ mice with Foxp3^YFP-Cre^ mice. We confirmed that the IFNAR was specifically deleted in CD4^+^Foxp3^+^ Tregs and not in CD4^+^Foxp3^-^ T cells, CD8^+^ T cells or B220^+^ B lymphocytes (S1A Fig). First, we compared the clearance of LCMV Armstrong from the sera of IFNAR^fl/fl^, IFNAR^fl/fl^ x Foxp3^YFP-Cre^, and IFNAR^-/-^ mice at early time points post-infection. We observed that IFNAR^fl/fl^ x Foxp3^YFP-Cre^ mice showed significantly higher viral titers (D3, D7 and D10) than IFNAR^fl/fl^ mice, and as expected IFNAR^-/-^ mice had the highest titers among the three groups [33, 38] (Fig 1A). However, IFNAR^fl/fl^ x Foxp3^YFP-Cre^ mice cleared LCMV Armstrong on day 14 post-infection.

This result is consistent with the studies of Srivastava et al. (2014) suggesting that the responses of the antiviral effector T cells early during LCMV Armstrong infection are compromised. Indeed, while both the frequencies and absolute numbers of CD8^+^CD44^+^ T cells specific for GP33-41 and NP396-404 did not differ between IFNAR^fl/fl^ and IFNAR^fl/fl^ x Foxp3^YFP-Cre^ mice on day 14 post-infection (Fig 1B and 1C), the production of the effector cytokines IFN-γ and TNF-α was markedly diminished (Fig 1E and 1F). While the frequency and absolute number of CD8^+^CD44^+^ T cells specific for GP276-286 were increased, the frequency of IFN-γ producing cells recognizing GP276-286 was still reduced (Fig 1D and 1G). We did observe a modest decrease in the frequency, but not absolute numbers of CD4^+^Foxp3^-^CD44^+^ T cells specific for LCMV GP66-76 (Fig 1H) and this was accompanied by a marked decrease in the production of both IFN-γ and TNF-α by the GP66-76 specific cells (Fig 1I).

### Lack of IFNAR signaling results in higher numbers of activated Treg during LCMV Armstrong infection

Taken together, these studies are consistent with the possibility that the absence of signaling via the IFNAR in Tregs during LCMV Armstrong infection potentiated their suppressive activity resulting in a failure to fully activate LCMV-specific T effector cells [39]. We observed that Treg cell numbers from infected WT and IFNAR^fl/fl^ x Foxp3^YFP-Cre^ mice on day 4 and day 5 are similar and that Treg cells from both the WT and IFNAR^fl/fl^ x Foxp3^YFP-Cre^ mice decrease similarly on day 7 post-infection (S1B Fig). While Srivastava et al (2014) reported a marked decrease in WT Foxp3^+^ Tregs on day 7 post infection in mixed bone marrow chimeras, we did not observe a decrease in Tregs on day 5 and the reduction in Treg frequencies and absolute numbers on day 7 was seen in both IFNAR^fl/fl^ and IFNAR^fl/fl^ x Foxp3^YFP-Cre^ mice (S1B Fig). In contrast, Foxp3^+^ Tregs frequencies and numbers were increased on day 14 post LCMV Armstrong infection in IFNAR^fl/fl^ x Foxp3^YFP-Cre^ mice. Most notably, the percentages and absolute numbers of activated Tregs were increased in IFNAR^fl/fl^ x Foxp3^YFP-Cre^ mice compared to IFNAR^fl/fl^ mice on day 5, 7 and 14 post Armstrong infection (S1C Fig). In addition to elevated levels of CD44, Tregs in IFNAR^flf/fl^ x Foxp3^YFP-Cre^ mice also expressed higher percentages of other activation markers, including Ki-67^+^, ICOS^+^ and TIGIT^+^ (S1D and S1E Fig), consistent with an activated phenotype and greater degree of proliferation at day 5 post Armstrong infection. We did not see any differences in the frequencies of activated CD4^+^Foxp3^-^ and CD8^+^ T cells (S1F Fig) in IFNAR^fl/fl^ x Foxp3^YFP-Cre^ mice.

### Absence of IFNAR signaling potentiates viral persistence in chronic LCMV infection

**Fig 2:**
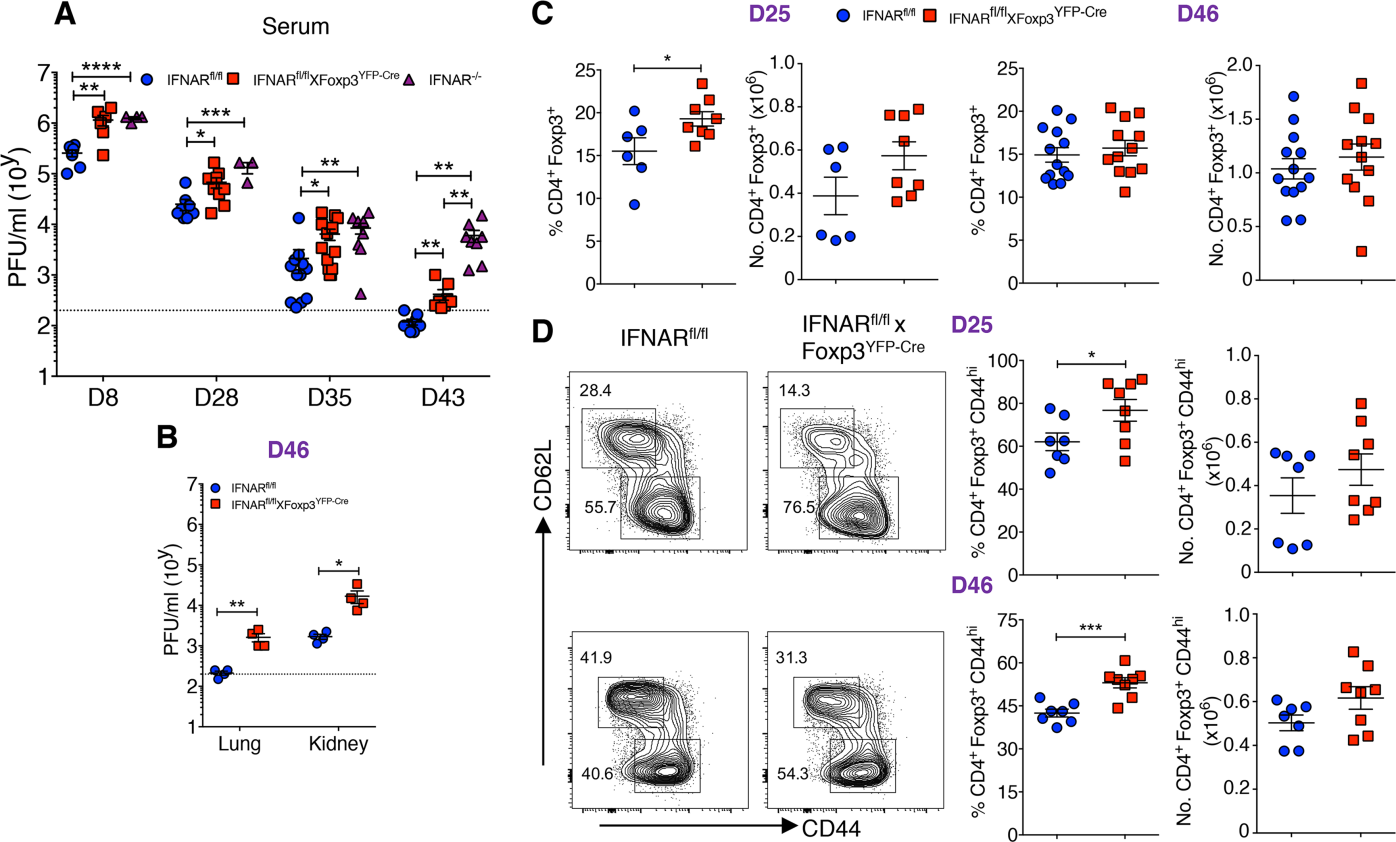
Absence of IFNAR signaling potentiates viral persistence in chronic LCMV infection. (**A**) LCMV titers were determined from Cl-13 infected IFNAR^fl/fl^, IFNAR^fl/fl^ x Foxp3^YFP-Cre^, and IFNAR^-/-^ mice sera on indicated days. (**B**) Lung and kidney tissues from day 46 Cl-13 infected IFNAR^fl/fl^ and IFNAR^fl/fl^ x Foxp3^YFP-Cre^ mice were assessed for detection of LCMV. (**C**) IFNAR^fl/fl^ and IFNAR^fl/fl^ x Foxp3^YFP-Cre^ mice spleen cells on day 25 and day 46 Cl-13 infection were analyzed for Foxp3^+^ Treg among CD4^+^ T cells. (**D**) CD44^+^ and CD62L^+^ cells were determined within gated Foxp3^+^ T cells on indicated days during chronic LCMV infection. * *P* < 0.05, ** *P* < 0.01, *** *P* < 0.001, and **** *P* < 0.0001 (unpaired two-tailed Student’s *t*-test). Data are shown from three to four experiments (**A**) involving three to fourteen mice, representative of two independent experiments (**B**) from four mice per group, and two to three experiments (**C** and **D**) on indicated days involving six to thirteen mice per group (Mean±SEM).

The role of Tregs in the maintenance of chronic viral infection and effector T cell exhaustion has been difficult to define as it has been technically challenging to specifically deplete Tregs without the induction of autoimmune disease [40–42]. To evaluate the role of IFNAR signaling in Tregs during persistent chronic viral infection, mice were infected with LCMV Cl-13. We initially examined viral titers by plaque assay in serum at different time points during infection. On days 8, 25, 35 and 43-post infection, IFNAR^fl/fl^ x Foxp3^YFP-Cre^ mice had significantly higher viral titers compared to IFNAR^fl/fl^ mice, and as expected IFNAR^-/-^ mice had significantly higher titers than IFNAR^fl/fl^ mice (Fig 2A). Notably, both the lungs and kidneys of IFNAR^fl/fl^ x Foxp3^YFP-Cre^ mice on day 46 post infection had significantly higher viral titers than IFNAR^fl/fl^ controls (Fig 2B). These data indicate that IFNAR deficiency specifically in Tregs enhances LCMV persistence.

The persistence of Cl-13 infection among IFNAR^fl/fl^ x Foxp3^YFP-Cre^ mice led us to examine the kinetics and activation of Tregs during chronic LCMV infection. The frequencies and absolute numbers of Tregs were higher in IFNAR^fl/fl^ x Foxp3^YFP-Cre^ mice on day 25-post infection, but not on day 46-post infection (Fig 2C). However, the activation state of the Tregs as measured by CD44 expression was higher on both days 25 and 46 in IFNAR^fl/fl^ x Foxp3^YFP-Cre^ mice compared to IFNAR^fl/fl^ mice (Fig 2D). In contrast, no significant differences were observed in the frequencies or absolute numbers of CD4^+^Foxp3^-^ or CD8^+^ T cells and their levels of CD44 expression on day 25 post-infection; however, on day 46 post-infection, the percentages of CD4^+^Foxp3^-^CD44^hi^ T cells were higher in IFNAR^fl/fl^ x Foxp3^YFP-Cre^ mice (S2A–S2D Fig). Furthermore, Cl-13 infected IFNAR^fl/fl^ x Foxp3^YFP-Cre^ mice exhibited greater morbidity as manifest by a greater reduction in body weight than IFNAR^fl/fl^ mice (S2E Fig). Taken together, these results demonstrate similar to acute infection, failure of signaling via the IFNAR in Tregs results in enhanced Treg activation accompanied by decreased viral clearance.

### Absence of IFNAR signaling in Tregs results in decreased virus-specific cytokine production and enhanced virus-specific T effector cell exhaustion

**Fig 3:**
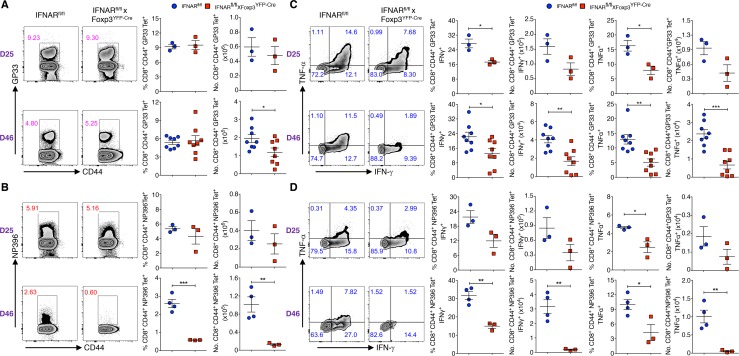
Absence of IFNAR signaling in Tregs results in decreased virus-specific CD8^+^ T cells and cytokine production. (**A** and **B**) Spleen cells of chronic LCMV infected (days 25 and 46) IFNAR^fl/fl^ and IFNAR^fl/fl^ x Foxp3^YFP-Cre^ mice were analyzed for GP33 Tet^+^ and NP396 Tet^+^ T cells within CD8^+^CD44^+^ T cells. (**C** and **D**) Spleen cells from (days 25 and 46) Cl-13 infected mice were stimulated with GP33, NP396, and Golgi stop for 5 hours at 37 ^o^C. Frequencies and absolute numbers of IFN-γ^+^ and TNF-α^+^ cytokine producing cells within CD8^+^CD44^+^GP33 Tet^+^ and CD8^+^CD44^+^NP396 Tet^+^ T cells are shown. * *P* < 0.05, ** *P* < 0.01, and *** *P* < 0.001 (unpaired two-tailed Student’s *t*-test). Data shown (**A-D**) from a representative and two experiments involving three to eight mice per group (Mean±SEM).

To determine if the enhanced Treg activation and diminished viral clearance in Cl-13 infected IFNAR^fl/fl^ x Foxp3^YFP-Cre^ mice results in decreased antiviral T effector cell responses, we examined the LCMV-specific responses of effector T cells. On day 25-post infection, both the absolute numbers of GP33 and NP396 tetramer positive T cells were similar (Fig 3A and 3B), while the frequencies of IFN-γ and TNF-α producing cells were lower (Fig 3C and 3D). However, on day 46-post infection, the absolute numbers of both antigen-specific CD8^+^ T cells were significantly decreased (Fig 3A and 3B) and this was accompanied by a marked decrease in the frequencies and absolute numbers of IFN-γ and TNF-α producing cells (Fig 3C and 3D). A moderate increase in the absolute number of CD4^+^Foxp3^-^CD44^+^GP66 Tet^+^ T cells frequencies was observed in the IFNAR^fl/fl^ x Foxp3^YFP-Cre^ mice on day 46-post infection, but not on day-25 post infection (S3A Fig). However, only low levels of IFN-γ and TNF-α were produced and no differences were observed in cytokine production by CD4^+^Foxp3^-^CD44^+^GP66 Tet^+^ T cells among IFNAR^fl/fl^ and IFNAR^fl/fl^ x Foxp3^YFP-Cre^ mice on both day 25 and 46-post infection (S3B Fig). Thus, decreased IFNAR signaling in Tregs resulted in reduced number and frequencies of CD8^+^ virus specific IFN-γ and TNF-α producing cells.

**Fig 4:**
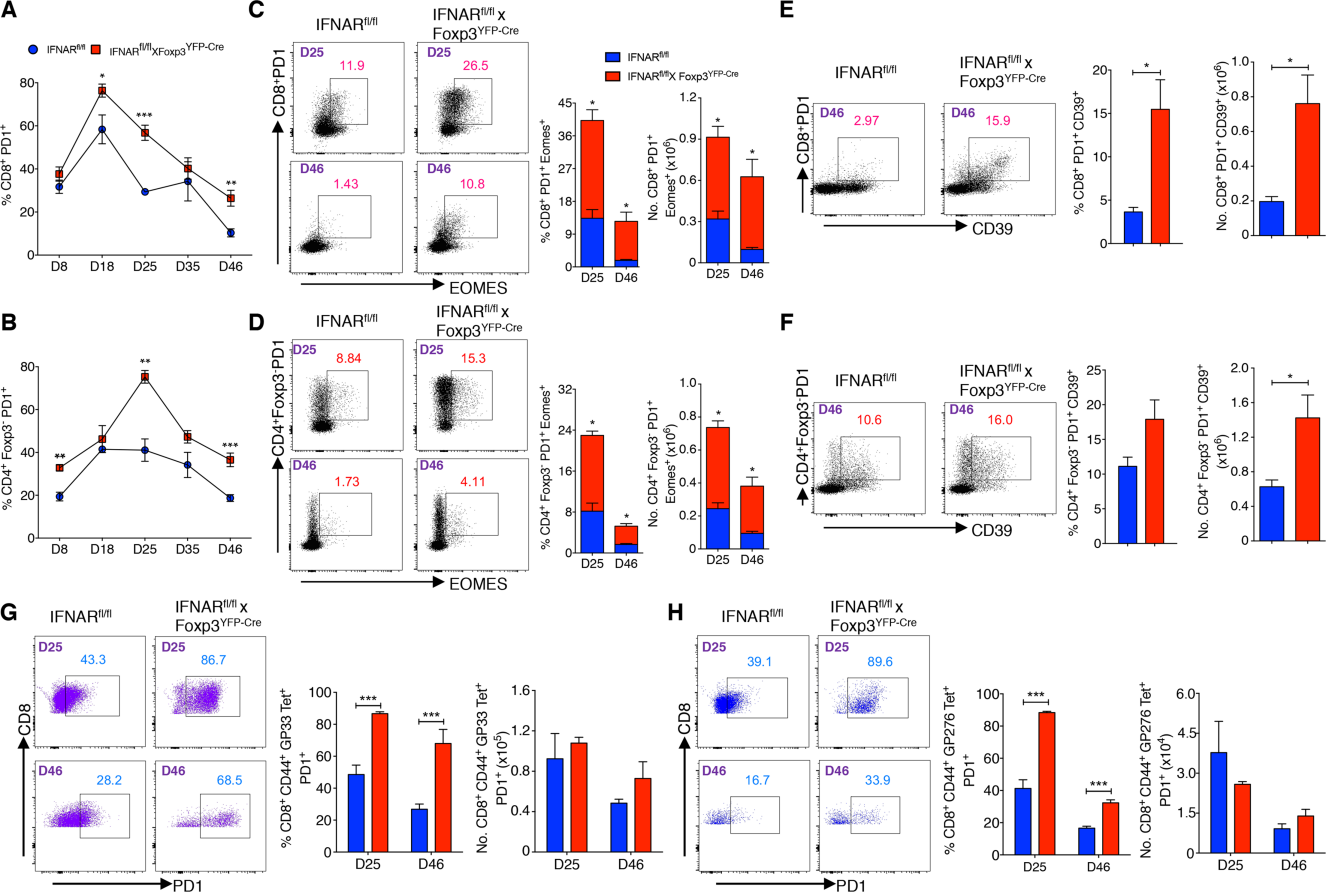
Absence of IFNAR signaling in Tregs results in enhanced virus-specific T cell exhaustion. (**A** and **B**) Kinetics of PD1 expression was shown on gated CD8^+^ and CD4^+^Foxp3^-^ T cells during chronic LCMV infection. (**C** and **D**) Percentage and total numbers of PD1^+^ EOMES^+^ cells among gated CD8^+^ and CD4^+^Foxp3^-^ T cells were plotted from days 25, and 46 Cl-13 infected mice. (**E** and **F**) PD1^+^CD39^+^ cells frequencies and numbers were estimated within CD8^+^ T and CD4^+^Foxp3^-^ T cells from day 46 Cl-13 infected mice. (**G** and **H**) PD1 expression was evaluated within CD8^+^CD44^+^GP33 and CD8^+^CD44^+^GP276 Tet^+^ T cells from Cl-13 infected mice on indicated days. * *P* < 0.05, ** *P* < 0.01, and *** *P* < 0.001 (unpaired two-tailed Student’s *t*-test). Data represent from two to five experiments (**A** and **B**), representative of two experiments (**C-H**), on indicated days with three to four mice per group in each experiment (Mean±SEM).

High levels of PD-1 expression are one of the hallmarks of T cell exhaustion. On both days 25- and 46-post infection, the frequency of PD1 expressing CD8^+^ and CD4^+^Foxp3^-^ T cells were higher in IFNAR^fl/fl^ x Foxp3^YFP-Cre^ mice (Fig 4A and 4B). Two other markers of T cell exhaustion, the transcription factor eomesodermin (EOMES), and CD39 can be co-expressed with PD-1 on exhausted T cells [43, 44]. Higher percentages and absolute numbers of EOMES^+^PD-1^+^ cells within the CD8^+^ and CD4^+^Foxp3^-^ populations were present in the IFNAR^fl/fl^ x Foxp3^YFP-Cre^ mice (Fig 4C and 4D). Correspondingly, PD1^+^CD39^+^ frequencies and total numbers were higher in gated CD8^+^ T cells from IFNAR^fl/fl^ x Foxp3^YFP-Cre^ mice (Fig 4E). While the frequencies of gated CD4^+^Foxp3^-^PD1^+^CD39^+^ T cells did not differ between IFNAR^fl/fl^ and IFNAR^fll/fl^ x Foxp3^YFP-Cre^ mice, the absolute numbers of CD4^+^Foxp3^-^PD1^+^CD39^+^ T cells were higher in Treg-specific IFNAR deficient mice (Fig 4F). Similarly, the frequencies of PD-1^+^ T cells were greater among gated CD8^+^CD44^+^GP33 and CD8^+^CD44^+^GP276 Tet^+^ T cells in IFNAR^fl/fl^ x Foxp3^YFP-Cre^ mice (Fig 4G and 4H). CD8^+^CD44^+^NP396 Tet^+^ and CD4^+^Foxp3^-^CD44^+^GP66 Tet^+^ populations from day 46 Cl-13 infected IFNAR^fl/fl^ x Foxp3^YFP-Cre^ mice also had higher proportions of PD1 expressing cells (S4A and S4B Fig). In addition, it has been demonstrated that Tregs with higher levels of PD1 expression can mediate enhanced suppression in LCMV infection [45], we also found that Tregs from Cl-13 infected IFNAR^fl/fl^ x Foxp3^YFP-Cre^ mice had significantly higher expression of PD1 than controls on days 25 and 35 post infection (S4C Fig). These data demonstrate that the enhanced Treg suppression seen in the absence of IFNAR signaling during Cl-13 infection results in markedly reduced cytokine production by virus-specific CD8^+^ T cells as well as a phenotype consistent with exhaustion.

### Absence of IFNAR signaling on Tregs results in defective generation of memory T cells in LCMV infection

**Fig 5:**
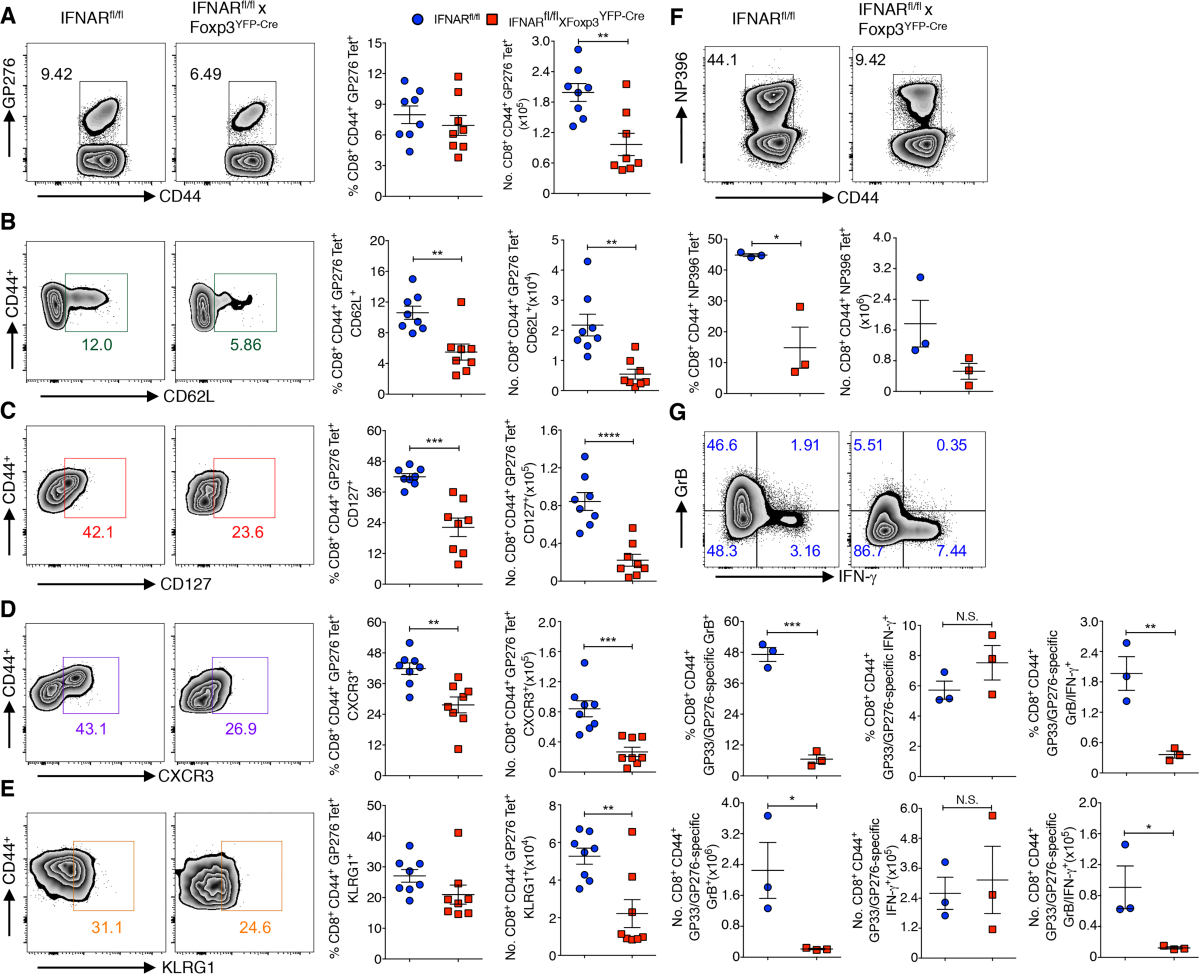
Absence of IFNAR signaling in Tregs results in defective generation of memory T cells in LCMV infection. (**A**) Spleen cells of chronic LCMV infected (day 46) IFNAR^fl/fl^ and IFNAR^fl/fl^ x Foxp3^YFP-Cre^ mice were analyzed for frequencies and absolute numbers of GP276 Tet^+^ T cells. Frequencies and absolute numbers of CD62L^+^ (**B**), CD127^+^ (**C**), CXCR3^+^ (**D**), and KLRG1^+^ (**E**) cells were determined within gated CD8^+^CD44^+^GP276 Tet^+^ T cells (as in **A**). Splenocytes harvested on day 5 Cl-13 recall challenge after day 30 LCMV Armstrong infection (memory phase), and CD8^+^CD44^+^ T cells were analyzed for frequencies and absolute numbers of NP396 Tet^+^ T cells (**F**). Spleen cells from Cl-13 recall challenge were stimulated with GP33, GP276, and Golgi stop for 5 hours at 37 ^o^C, frequencies and absolute numbers of CD8^+^CD44^+^ T cells producing Granzyme B, and IFN-γ were analyzed (**G**). * *P* < 0.05, ** *P* < 0.01, *** *P* < 0.001, and **** *P* < 0.0001 (unpaired two-tailed Student’s *t*-test). Data derived from one to two experiments involving three to eight mice per group (Mean±SEM).

Exhausted CD8^+^ T cells have reduced memory cell potential which is secondary to higher LCMV antigen persistence in infected mice [46]. To determine whether the enhanced T cell exhaustion phenotype observed in IFNAR^fl/fl^ x Foxp3^YFP-Cre^ mice is associated with a reduction in the formation of virus-specific memory T cells, we examined the levels of expression of three memory cell makers (CD62L, CD127, CXCR3) on gated CD8^+^CD44^+^ GP276 Tet^+^ T cells (Fig 5A). The frequencies and the absolute numbers of all three memory populations were reduced in IFNAR^fl/fl^ x Foxp3^YFP-Cre^ mice on day 46 post infection compared to IFNAR^fl/fl^ control mice (Fig 5B–5D). Similar results were seen within the CD8^+^CD44^+^GP33 Tet^+^ population (S5A–S5D Fig). While the number of memory CD8^+^ T cells is usually inversely correlated with terminally differentiated T cells as measured by KLRG-1 expression [47], the frequencies and absolute numbers of CD8^+^CD44^+^GP276/GP33 Tet^+^ KLRG-1^+^ T cells were also lower in IFNAR^fl/fl^ x Foxp3^YFP-Cre^ mice (Fig 5E and S5E Fig). We further examined the protective capacity of memory CD8^+^ T cells by re-infecting the day 30 Armstrong infected mice with Cl-13 virus. On day 5 post Cl-13 infection, IFNAR^fl/fl^ x Foxp3^YFP-Cre^ mice had reduced frequencies and numbers of CD8^+^CD44^+^NP396 Tet^+^ cells, and in addition GP33- and GP276-stimulated CD8^+^CD44^+^ T cells from IFNAR^fl/fl^ x Foxp3^YFP-Cre^ infected mice produced significantly less Granzyme B (GrB) positive and GrB/IFN-γ double positive cells compared to CD8^+^ T cells from control mice (Fig 5F and 5G), however IFN-γ positive cells are tended to be more in infected IFNAR^fl/fl^ x Foxp3^YFP-Cre^ mice but they are not significant compared to control mice. Collectively, these results demonstrate that the higher load of virus is associated with a defect in the generation of virus-specific memory T cells in the absence of IFNAR signaling in Tregs.

### Transcriptional profiling of Tregs during Armstrong and Cl-13 infection

**Fig 6:**
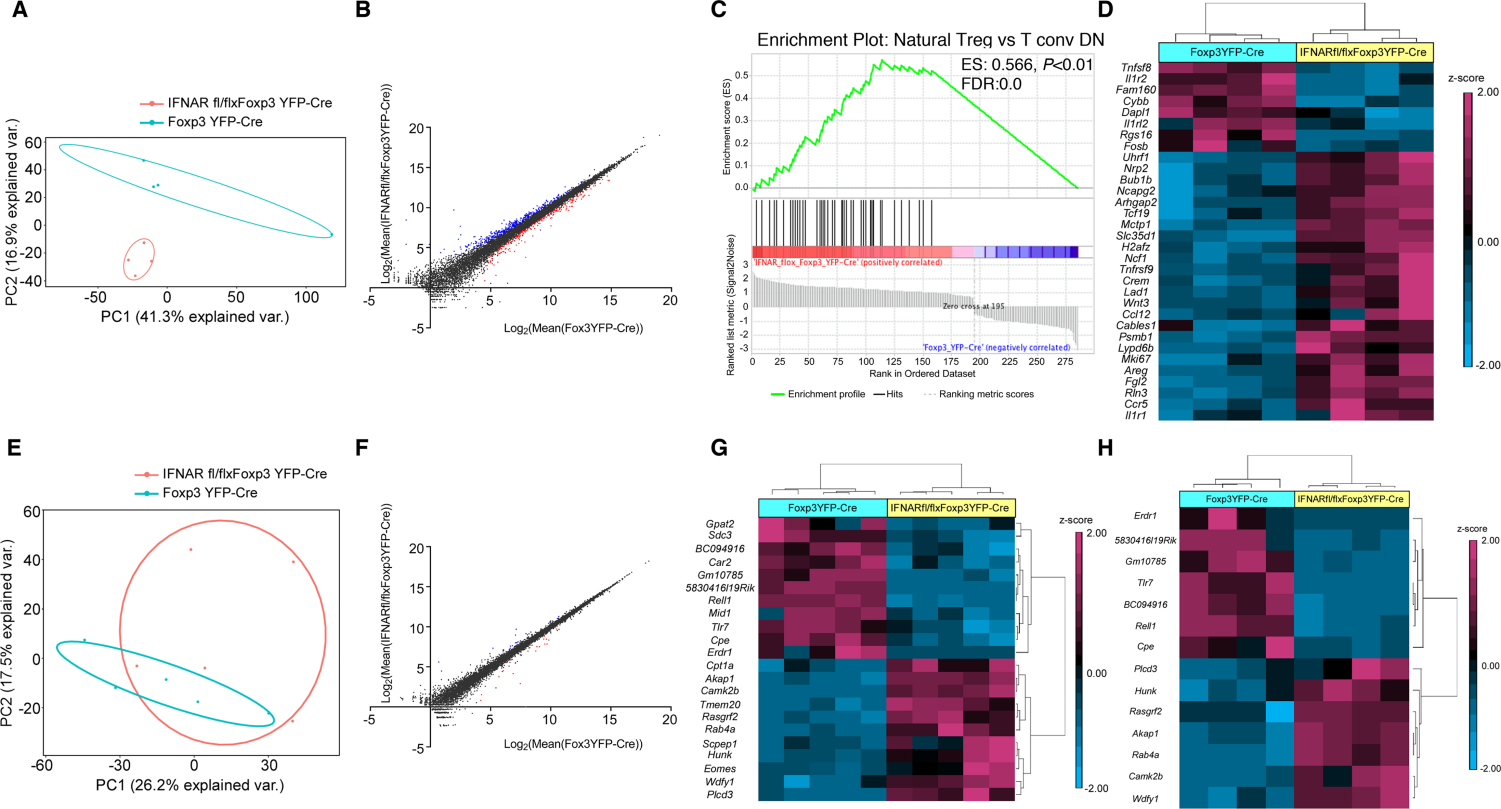
Transcriptome analysis of Foxp3^+^ Tregs from LCMV infected Treg-specific IFNAR-deficient mice. (**A**) PCA was performed on day 5 LCMV Armstrong infected Foxp3^YFP-Cre^ and IFNAR^fl/fl^ x Foxp3^YFP-Cre^ mice sorted CD4^+^YFP^+^ Treg cells RNA-seq samples (4 samples in each group). (**B**) Scatter plot showing the comparison of global gene expression profiles of Tregs between Armstrong infected Foxp3^YFP-Cre^ and IFNAR^fl/fl^ x Foxp3^YFP-Cre^ mice. Total of 586 genes were significantly differentially expressed and colored, 249 genes (red) were down regulated, and 337 genes (blue) upregulated in IFNAR^fl/fl^ x Foxp3^YFP-Cre^ mice (fold change 1.5 and above, adjusted *P* < 0.05). (**C**) GSEA for non-IFN related genes, showing enrichment plot for natural Treg vs. T conv DN gene set (36 out 42 genes were enriched in core, Enrichment score: 0.566, *P* < 0.01, FDR: 0.0) with positive enrichment in IFNAR^fl/fl^ x Foxp3^YFP-Cre^ mice Tregs relative to Foxp3^YFP-Cre^ mice Tregs. (**D**) Heat map showing the significant differential expression of 32 Treg-signature genes (fold change 1.5 and above, adjusted *P* < 0.05), as previously reported [49, 50], differentially expressed genes were normalized by z-score. (**E**) PCA was performed on day 25 LCMV Cl-13 infected Foxp3^YFP-Cre^ and IFNAR^fl/fl^ x Foxp3^YFP-Cre^ mice sorted CD4^+^YFP^+^ Treg cells RNA-seq samples (5 samples in each group). (**F**) Scatter plot showing the comparison of global gene expression profiles of Tregs between Cl-13 infected Foxp3^YFP-Cre^ and IFNAR^fl/fl^ x Foxp3^YFP-Cre^ mice. Total of 36 genes were significantly differentially expressed and colored, 23 genes (red) were down regulated, and 13 genes (blue) upregulated in IFNAR^fl/fl^ x Foxp3^YFP-Cre^ mice (fold change 1.5 and above, adjusted *P* < 0.05). (**G**) Heat map showing the significant differential expression of 22 non-IFN related genes (fold change 1.5 and above, adjusted *P* < 0.05, normalized by z-score). (**H**) Heat map showing the significant differential expression of 14 genes from LCMV Armstrong infected Foxp3^YFP-Cre^ mice and IFNAR^fl/fl^ x Foxp3^YFP-Cre^ mice Tregs, which were in consistent with transcriptome from LCMV Cl-13 infected mice Tregs (fold change 1.5 and above, adjusted *P* < 0.05, normalized by z-score). Armstrong infection transcriptome data obtained from an experiment involving four mice per group (**A-D** and **H**), and transcriptome data from Cl-13 infection, involved an experiment with five mice per group (**E-G**).

In order to better understand the molecular basis for the enhanced activation and suppressive function of Tregs from Treg-specific IFNAR-deficient mice during acute and chronic LCMV infection, we performed high-throughput RNA sequencing on sorted CD4^+^YFP^+^ Tregs isolated from day 5 Armstrong-infected Foxp3^YFP-Cre^ and IFNAR^fl/fl^ x Foxp3^YFP-Cre^ mice. Principal component analysis (PCA) showed distinct clustering of Tregs from Foxp3^YFP-Cre^ mice relative to Tregs from IFNAR^fl/fl^ x Foxp3^YFP-Cre^ mice (Fig 6A). A total of 586 genes were significantly differentially expressed (249 genes were down, and 337 genes were up) in IFNAR^fl/fl^ x Foxp3^YFP-Cre^ mice (fold change 1.5 and above, adjusted *P* < 0.05) (Fig 6B). Among the 586 genes, 174 genes were identified in the interferome database [48] (interferome.its.monash.edu.au) as IFN-signaling related (fold change 1.5 and above, adjusted *P* < 0.05) (S6A Fig), and were excluded from further analysis. We elected to exclude IFNAR regulated genes in order to perform an unbiased downstream analysis, as IFNAR signaling regulates the transcription of up to 2000 genes. The remaining 412 differentially expressed genes were exclusively non-IFN related. Of these, 156 genes were upregulated in infected Foxp3^YFP-Cre^ mice, and 256 genes were upregulated in infected IFNAR^fl/fl^ x Foxp3^YFP-Cre^ mice. Gene set enrichment analysis (GSEA) of the 412 non-IFN related genes revealed that the natural Treg vs conventional T cell gene set was enriched to a greater extent in IFNAR^fl/fl^ x Foxp3^YFP-Cre^ mice [36 genes out of 42 were in core enrichment, Enrichment score (ES): 0.566, *P* < 0.01, FDR:0.0] (Fig 6C). We observed that 32 out of 412 non-IFN related genes were differentially expressed (fold change 1.5 and above, adjusted *P* < 0.05, genes normalized by z-score; 24 genes in IFNAR^fl/fl^ x Foxp3^YFP-Cre^ mice and 8 genes in Foxp3^YFP-Cre^ mice were upregulated) and could be classified as Treg-signature genes as previously reported [49, 50] (Fig 6D). Representative upregulated genes in IFNAR^fl/fl^ x Foxp3^YFP-Cre^ mice include *Areg*, *Arhpag20*, *Bub1b*, *Ccl12*, *Ccr5*, *Il1r1*, *Mki67* (Ki67), *Ncf1*, *Nrp2*, *Tnfrsf9* (CD137), *Tcf19*, *Uhrf1*, and *Wnt3*; representative downregulated genes in IFNAR^fl/fl^ x Foxp3^YFP-Cre^ include *Cybb*, *Dapl1*, *Fam160a1*, *Il1r2*, and *Tnfsf8* (CD153). Some of the above upregulated genes from Tregs include *Areg*, *Ccl12*, *Mki67*, *Ncf1*, *Tnfrsf9*, *Uhrf1* are well characterized to modulate the enhanced Treg suppressive and proliferative function [49, 50]. Further, we also performed ingenuity pathway analysis (IPA) for non-IFN related genes which resulted in 45-top canonical pathways (adjusted p value < 0.1) (S6B Fig). Specifically, cell cycle: G2/M DNA damage checkpoint regulation pathway (genes involved: *Aurka*, *Ccnb2*, *Cdk1*, and *Top2a*), cyclins and cell cycle regulation pathway (genes involved: *Ccnb2*, *Ccne2*, *Cdk1*, and *E2f1*) are enriched positively in IFNAR^fl/fl^ x Foxp3^YFP-Cre^ Tregs compared to Tregs from Foxp3^YFP-Cre^ mice, suggesting that cell cycle genes are more functional in Treg-specific IFNAR deficient mice. Furthermore, the c-AMP mediated signaling pathway (genes involved: *Akap1*, *Camk2b*, *Chrm4*, *Crem*, *Fpr1*, *Prkar1b*, and *Ptger3*) is also enriched positively in IFNAR deficient Tregs. Through IPA, we analyzed non-IFN related genes for top networks based on co-expression, transcription factor binding site predictions and protein-protein interactions. The top two networks identified included cell cycle, DNA replication, recombination, repair, cancer; and cellular movement, hematological system development and function and immune cell trafficking (S6C Fig), differential expression of these associated genes in the pathways are shown (S1 and S2 Tables). Some of the associated genes in the networks include, transcription factors: *Depdc1*, *E2f1*, *E2f8*, *Foxm1*, *Mybl2*, and *Uhrf1* are downregulated, and *pydc4/Ifi16* (interferon gamma inducible protein 16) is upregulated in Tregs from Foxp3^YFP-Cre^ mice; cell cycle kinases: *Ccnb2*, *Aurkb*, and *Chek1* are also downregulated in Foxp3^YFP-Cre^ mice Tregs; immune cell genes such *Tnfrsf9* (CD137), *Ccl2*, *Ccr5*, and *Il1r1* are downregulated, in contrast *C5ar1*, *CD19*, *Il1r2*, *Itga2b* (CD41), *Ly6c1*, and *Tlr7* are upregulated in Foxp3^YFP-Cre^ mice Tregs. We also tested whether the reduced cell cycle gene signature in Tregs from Foxp3^YFP-Cre^ mice contributed to a greater degree of apoptosis, but Active caspase-3 staining showed no significant increase in the staining of Tregs from day 5 Armstrong infected Foxp3^YFP-Cre^ mice compared to Tregs from IFNAR^fl/fl^ x Foxp3^YFP-Cre^ mice (S6D Fig).

In parallel, we also performed RNA sequencing on Tregs from day 25 post LCMV Cl-13 infection. PCA showed less distinct clustering (Fig 6E), and surprisingly, only 36 genes were significantly differentially expressed (23 genes were downregulated, and 13 genes were upregulated in IFNAR^fl/fl^ x Foxp3^YFP-Cre^ mice, fold change 1.5 and above, adjusted *P* < 0.05) in Tregs from Foxp3^YFP-Cre^ and IFNAR^fl/fl^ x Foxp3^YFP-Cre^ mice (Fig 6F). Among those differentially expressed genes, 14 genes were identified in the interferome database [48] as IFN-signaling related (fold change 1.5 and above, adjusted *P* < 0.05) (S7A Fig). Of the remaining 22 genes, 11 genes were upregulated in their expression in Tregs from Foxp3^YFP-Cre^ mice and IFNAR^fl/fl^ x Foxp3^YFP-Cre^ mice, respectively (Fig 6G). Additionally, IPA for non-IFN related genes resulted in 18-top canonical pathways (adjusted p value < 0.1) (S7B Fig), importantly, c-AMP mediated signaling pathway (genes involved: *Akap1*, and *Camk2b*) is enriched positively in Cl-13 infected IFNAR^fl/fl^ x Foxp3^YFP-Cre^ mice Tregs, this was shown similar pattern in Armstrong infected IFNAR^fl/fl^ x Foxp3^YFP-Cre^ mice. One of the top networks for non-IFN related genes include lipid metabolism, molecular transport and small molecule biochemistry (S7C Fig). Few of the associated genes in the network include, kinases: *Camk2b*, and *Hunk* and cytoplasmic enzymes: *Cpta1*, and *Scpep1* are down regulated, while proapoptotic factor *Erdr1* is upregulated in Foxp3^YFP-Cre^ mice Tregs.

We identified fourteen genes (fold change 1.5 and above, adjusted *P* < 0.05, normalized by z-score) differentially expressed in Tregs from LCMV Armstrong infected mice which were similarly differentially expressed in Tregs from LCMV Cl-13 infected mice including 7 that were up- and 7 down-regulated (Fig 6G and 6H). We further validated the differential expression of some of these non-IFN related genes during chronic infection which are common to both infection model or unique to chronic infection alone by quantitative real-time PCR (S7D Fig). Several of the upregulated genes include a-kinase anchoring protein 1 (*Akap1*), calcium/calmodulin-dependent protein kinase II b (*Camk2b*), Hormonally upregulated Neu-associated kinase (*Hunk*), *Rab4a* and *Rasgrf2*. Akap1 is associated with cAMP signaling, and it can act as a gap junction protein in facilitating the transfer of cAMP from Treg to effector T cells leading to inhibition of T cell receptor (TCR) signaling [51, 52]. Furthermore, *CamK2b*, *Hunk*, *Rab4a* and *Rasgrf2* have also been implicated in enhancement of Treg function [53–57]. Taken together, all these upregulated genes participate in heightened Treg suppressive function observed in the Tregs from IFNAR^fl/fl^ x Foxp3^YFP-Cre^ mice during both acute and chronic LCMV infections.

### Treg-specific IFNAR deficiency leads to an impaired antitumor immune response

**Fig 7:**
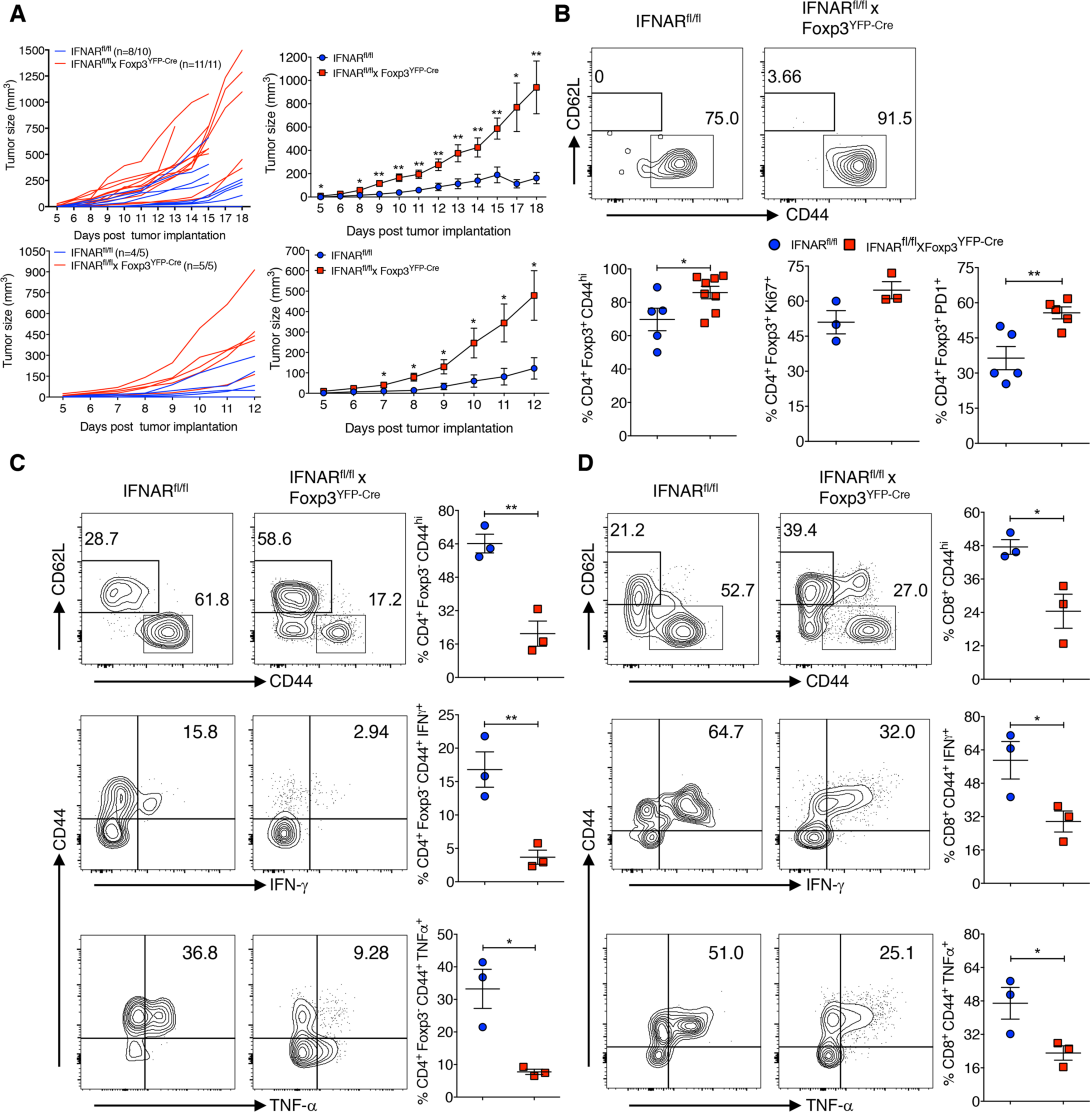
Treg-specific IFNAR deficiency leads to impaired antitumor immune response. (**A**) IFNAR^fl/fl^ and IFNAR^fl/fl^ x Foxp3^YFP-Cre^ mice were injected with 2x10^5^ MC38 cells (left panels) and or 1.25x10^5^ B16.F10 cells (right panels) subcutaneously, tumor growth in individual mice (left panels) and in pooled mice (two individual experiments, right panels) tumor volumes were plotted. (**B**) TIL were isolated on day 18 of MC38 tumor burden and determined CD44^+^, Ki-67^+^ and PD1^+^ cells within gated CD4^+^Foxp3^+^ T cells. TIL from day 18 MC38-post tumor implants were stained for CD44^+^ within CD4^+^Foxp3^-^ (**C**) and CD8^+^ T cells (**D**), and TIL were stimulated with cell stimulation cocktail containing PMA, ionomycin, Brefeldin A and monensin for 5 hours at 37 ^o^C. Frequencies of IFN-γ^+^ and TNF-α^+^ cytokine producing cells within the gated CD4^+^Foxp3^-^CD44^+^ and CD8^+^CD44^+^ T cells are shown. * *P* < 0.05, and ** *P* < 0.01 (unpaired two-tailed Student’s *t*-test). Data shown from two individual experiments representing five to eleven mice per group (**A**), and a representative of two independent experiments (**B-D**) involving three to eight mice per group (Mean±SEM).

Enhanced Treg cell function is very well documented in various tumor models and it has been associated with a poor prognosis [6]. To determine whether the absence of IFNAR signaling was associated with enhanced Treg suppression in a non-infectious setting, we utilized the mouse colon adenocarcinoma MC38 and mouse B16.F10 melanoma models. IFNAR^fl/f^ x Foxp3^YFP-Cre^ mice showed higher tumor incidence (MC38: n = 11/11, 100%; B16.F10: n = 5/5, 100%) and significantly increased volume than IFNAR^fl/fl^ mice (MC38: n = 8/10 mice, 80%, B16.F10: n = 4/5, 80%) (Fig 7A, left and right panels). Tregs and CD4^+^Foxp3^-^ cells isolated from tumor infiltrating lymphocytes (TIL) showed comparable frequencies in both strains of mice, however CD8^+^ T cell frequencies from TIL of IFNAR^fl/fl^ x Foxp3^YFP-Cre^ mice were increased. Nevertheless, both CD4^+^Foxp3^-^ and CD8^+^ T cells from IFNAR^fl/fl^ x Foxp3^YFP-Cre^ mice tended to proliferate less as assayed by Ki-67 expression (S8A–S8C Fig). Importantly, Tregs from IFNAR^fl/fl^ x Foxp3^YFP-Cre^ mice TIL expressed significantly higher levels of CD44, enhanced proliferation and expression of PD-1 (Fig 7B). Conversely, both CD4^+^Foxp3^-^ and CD8^+^ TIL from IFNAR^fl/fl^ x Foxp3^YFP-Cre^ mice expressed lower levels of CD44 and markedly reduced levels of IFN-γ and TNF-α production compared to TIL from IFNAR^fl/fl^ mice (Fig 7C: gated on CD4^+^Foxp3^-^ TILs and Fig 7D: gated on CD8^+^ TILs). These data strongly suggest that Tregs in TIL from IFNAR^fl/fl^ x Foxp3^YFP-Cre^ mice have enhanced suppressor activity in the tumor microenvironment and the phenotype of these Tregs within TIL closely resembles the activated hypersuppressive phenotype observed during acute and chronic LCMV infections.

## Discussion

Tregs mediate a multifaceted role in modulating the immune response to acute and chronic infectious agents. While their beneficial effects in decreasing immune pathology during the resolution phase of many infections is clear, Tregs can also mediate immune suppression resulting in pathogen persistence. During viral infections, rapid activation of the innate immune system generates inflammatory signals that can initially control the infection and ultimately influence the quality and magnitude of the adaptive antiviral effector T cell response. The best characterized innate inflammatory signals are the type I IFNs. During LCMV infection type I IFNs are produced in large quantities immediately following viral infection by plasmacytoid DCs as well as virus infected cells and primarily exert their effect on CD8^+^ T cells by extending their survival. In this report, we have demonstrated that type I IFNs can also exert beneficial effects by acting on Tregs to down-modulate their suppressive functions both early during the course of acute LCMV Armstrong infection and also later during virus persistence in chronic Cl-13 infection.

Srivastava et al. (2014), have previously examined the effects of type I IFN on Tregs during the course of acute LCMV infection. They concluded that type I IFNs down-modulated Treg function but postulated that the effects of type I IFNs were secondary to a selective decrease in the number of highly suppressive effector Tregs, and secondary to the pro-apoptotic and anti-proliferative actions of type I IFNs early in the course of infection. The major difficulty in the interpretation of this study is that the experimental model they used did not allow them to selectively examine the effects of IFNs on Tregs in the absence of its effects on other cell types. Similarly, one previous study also showed that Tregs were reduced in infected wild type compared to control mice during first week post LCMV-Docile infection, but Tregs expanded to a greater extent from the second weeks onwards. Treg expansion was more pronounced in IL-21R deficient mice, suggesting IL-21 signaling restricts proliferation of Tregs during LCMV infection [58]. The availability of mice with a conditional deletion of the IFNAR in Tregs allowed us to dissect the mechanistic basis of IFNAR signaling in Tregs resulting in their reduced suppressive function and in more efficient antiviral and antitumor immune responses.

Similar to the previous study [39], we found that the generation of antigen-specific CD8^+^ and CD4^+^ T cells was comparable in Treg-specific IFNAR-deficient mice and WT controls during acute LCMV infection, but that both the virus-specific CD8^+^ and CD4^+^ T cells in IFNAR^fl/fl^ x Foxp3^YFP-Cre^ mice produced markedly reduced amounts of IFN-γ and TNF-α accompanied by a slower rate of viral clearance than the controls. Most importantly, we did not observe a decrease in the percentages or absolute numbers of Tregs in the WT control mice that differed from IFNAR^fl/fl^ x Foxp3^YFP-Cre^ mice on day 4 or 5 post-infection. We did detect a decrease in Tregs in the IFNAR^fl/fl^ X Foxp3^YFP-Cre^ mice on day 7 post-infection, but we observed an identical decrease in Tregs in the control IFNAR^fl/fl^ mice. In contrast to the loss of memory/effector Tregs observed in WT mice by Srivastava et al. (2014), we observed an enhanced percentage of memory/effector Treg as defined by CD44 expression on days 5, 7 and 14 post-infection in IFNAR^fl/fl^ x Foxp3^YFP-Cre^ mice, as well as higher levels of expression of Ki-67, ICOS and TIGIT. Taken together, our results are most consistent with an enhanced suppressive phenotype of Tregs in the absence of IFNAR signaling in acute virus infection albeit the mice ultimately cleared the infection.

We observed a similar but more profound suppressive phenotype in IFNAR^fl/fl^ x Foxp3^YFP-Cre^ mice following Cl-13 infection, as the mice had higher serum titers of virus and also had higher viral titers in lungs and kidneys for as long as 46 days post infection. We observed decreased numbers of antiviral antigen-specific CD8^+^ T cells accompanied by a profound decrease in effector cytokine production. Increased viral persistence resulted in marked expression of markers associated with T cell exhaustion (PD-1, CD39, EOMES) [43, 44, 46] and decrease in generation of antigen-specific memory CD8^+^ T cells. The decrease in the formation of memory T cells in IFNAR^fl/fl^ x Foxp3^YFP-Cre^ mice in Cl-13 infection should be contrasted to the effects of IL-10 producing Treg cells in augmenting memory T cell formation following Armstrong infection [47]. Slight increases in both the percentages, but not the absolute numbers of Tregs were seen on days 25 and 46 post infection. However, at both times points, we observed a significant increase in the percentages of activated/effector Tregs. To determine if the hyperactivated/hypersuppressive phenotype observed in the absence of IFNAR signaling in Tregs was unique to viral infections, we also examined the responses of IFNAR^fl/fl^ x Foxp3^YFP-Cre^ mice to transplantable tumor models. Markedly enhanced growth of the tumor was observed in these mice accompanied by an enhanced percentage of activated PD-1^+^ tumor infiltrating Tregs. In addition, the activation and cytokine production by both CD4^+^Foxp3^-^ and CD8^+^ tumor infiltrating T cells were markedly suppressed. Taken together, these studies demonstrate that IFNAR signaling in Tregs plays a critical role in down-modulating, but certainly not abolishing, their suppressive function and may in viral infections orchestrate the balance between immunopathology and eradication of the virus.

To begin to elucidate the mechanistic basis for the suppressive function of IFNAR-deficient Tregs during both acute and Cl-13 infection, we performed high-throughput RNA sequencing of Foxp3^+^ Tregs from both controls and IFNAR^fl/fl^ x Foxp3^YFP-Cre^ mice on day 5 Armstrong and day 25 Cl-13 infection. A group of Treg-signature genes (*Areg*, *Arhpag20*, *Bub1b*, *Ccl12*, *Ccr5*, *Il1r1*, *Mki67* (Ki67) *Ncf1*, *Nrp2*, *Tnfrsf9*, *Tcf19*, *Uhrf1*, and *Wnt3*) were expressed at higher levels in IFNAR^fl/fl^ x Foxp3^YFP-Cre^ mice than WT controls on day 5 Armstrong infection. These genes have previously been identified as Treg-Up signature genes [49, 50] and their enhanced expression is consistent with the hyperactivated phenotype of the Tregs at that time point. We did not observe upregulation of this group of genes on day 25 of Cl-13 infection. Interestingly, both in Armstrong and Cl-13 infection, we observed the differential expression of a number of genes in IFNAR^fl/fl^ x Foxp3^YFP-Cre^ Tregs which might also play a role in their enhanced suppressive function including *Akap1*, *Camk2b*, *Rasgrf2* and *Hunk*. Akap1, which serves as a gap junction protein, is involved in transferring pools of cAMP from Treg to effector T cells resulting in inhibition of TCR signaling [51, 52]. Camk2b participates in the activation of nuclear factor kappa-B, which in turn plays a role in Treg development by stabilizing Foxp3 [53, 54]. Both Camk2b and Hunk have been shown to be have higher levels of expression in human Tregs than conventional effector T cells [55]. Lastly, Rasgrf2 is involved in stimulation of TCR signaling through activation of nuclear factor for activated T cells (NF-AT) [57]. Conversely, we also observed group of genes (*Erdr1*, *Rell1*, *Tlr7*) whose expression is downregulated in Tregs from both IFNAR^fl/fl^ x Foxp3^YFP-Cre^ Armstrong and Cl-13 infected mice. These genes have been described as potentially playing a role in apoptosis [59–61] and thus may be involved in the reduced suppressive function of Tregs after IFNAR signaling. We did not observe any differences between IFNAR^fl/fl^ x Foxp3^YFP-Cre^ and WT mouse Tregs in expression of Active caspase-3 on day 5 post Armstrong infection again consistent with our data that cell death is not playing a role in reduced Treg suppression in WT mice, although, we cannot exclude the involvement of other cell death pathways. Future studies involving over expression and/or deletion of these genes in Tregs will be needed to specifically implicate one or several of these genes in Treg-mediated suppression during viral infections. We have not yet performed similar gene expression studies in IFNAR sufficient and deficient Tregs derived from the tumor microenvironment and compared such data with Tregs from LCMV-infected mice.

Our study demonstrates that one of the multiple cellular targets of Type I IFNs during viral infection are Treg cells and that the functional result of this interaction is a downregulation of Treg suppressor function. A similar process may take place in the tumor microenvironment and may be responsible for some of the antitumor effects of this group of cytokines [62, 63]. Thus, type I IFNs should be added to the long list of cytokines (IL-1β, IL-4, IL-6, IL-15, IL-21) [64–72] and members of the tumor necrosis factor superfamily (TNFSF) (GITR-L, 4-1BB-L, OX40-L and TNF-α) [73–76] that are purported to decrease Treg suppressive function in autoimmune and infectious disease models. However, the abrogation of suppression is more frequently mediated by the action of the cytokine or TNFSF member on the responder T cells resulting in resistance to suppression [73, 77], whereas we have definitively demonstrated that Tregs are the targets cells in this model. While type I IFNs may attenuate Treg suppression, it remains clear that the major and undefined cellular target for the immunosuppressive effects of type I IFNs in chronic LCMV infection is not the Tregs, as IFNAR^fl/fl^ x Foxp3^YFP-Cre^ mice have elevated viral titers, while LCMV Cl-13 infected mice treated with a neutralizing antibody against IFNAR have decreased viral titers [33, 34]. Thus, an approach to target IFNAR in normal hosts for inhibition of Treg suppressive function in chronic infection or in cancer would be difficult.

## Materials and methods

### Ethics statement

This study was carried out in strict accordance with the recommendations for the Care and Use of Laboratory Animals of the National Institutes of Health. The protocol was approved by National Institute of Allergy and Infectious Diseases Animal Care and Use Committee (Protocol No: LI-5E).

### Mice

Foxp3^YFP-Cre^ (expressing Cre recombinase regulated by Foxp3 promoter) mice were purchased from Jackson Laboratories (Bar Harbor, ME). IFNAR^-/-^ mice were obtained by National Institute of Allergy and Infectious Diseases (NIAID), and maintained in Taconic Farms (Germantown, NY). IFNAR^fl/fl^ mice were generously provided by Ulrich Kalinke (Paul-Ehrlich Institut, Langen, Germany), and crossed with Foxp3^YFP-Cre^ mice to generate Treg-lineage specific IFNAR-deficient mice. All strains of mice used in this study were age 8–12 weeks of age), gender matched, and bred in-house.

### LCMV infection and plaque assay

LCMV Armstrong and Cl-13 viruses (Shevach Laboratory) were propagated in baby hamster kidney-21 fibroblast cells [American Type Culture Collection (ATCC), Manassas, VA]. Viral titers were determined by plaque assay using Vero African-green-monkey kidney cells (ATCC). Viral stocks were frozen at -80 ^o^C until use. Mice were infected with the diluted virus in 1x sterile phosphate buffer saline (PBS) (Armstrong virus, 2x10^5^ plaque forming unit (pfu)/mouse, i.p., or Cl-13 virus, 2x10^6^ pfu/mouse, i.v.). LCMV titers in sera and organs were determined by plaque assay using Vero cells as described [78].

### Lymphocytes isolation

Spleens were harvested from naive (uninfected) and infected mice on indicated days and homogenized the tissues using cell strainer (70 μm, Nest Scientific USA, Rahway, NJ). Red blood cells were lysed using sterile ACK lysing buffer [NH_4_Cl (0.15 M), KHCO_3_ (10 mM) and Na_2_EDTA (0.1 mM), pH 7.3]. Lymphocytes were washed, suspended in sterile complete medium [RPMI medium supplemented with 10% heat-inactivated fetal bovine serum (FBS), L-glutamine (2 mM), sodium pyruvate (1 mM), HEPES (1 mM), non-essential amino acids (0.1 mM), 2-mercaptoethanol (50 μM), and penicillin and streptomycin (100 U/ml)], and total live cells were counted.

### Cell surface, intracellular and tetramer staining

Cell surface staining was performed as described [79]. Briefly after harvest, spleen cells (3x10^6^ cells) or tumor infiltrating lymphocytes (TIL) were suspended in sterile complete medium. For surface staining, cells in staining buffer (PBS, 10% heat-inactivated FBS, and 0.05% sodium azide) were incubated for 30 min at 4 ^o^C, then stained with the following surface murine conjugate antibodies: anti-CD4, anti-CD62L, anti-CD39, anti-CD127, anti-IFNAR1, anti-B220 (all are from eBioscience, San Diego, CA); anti-CD8a, anti-CD44, anti-TIGIT (all are from BD Biosciences, San Jose, CA); anti-PD1, anti-KLRG1, anti-CXCR3, and anti-ICOS (all are from BioLegend, San Diego, CA) and live/dead fixable aqua dead cell stain kit (Life Technologies, Carlsbad, CA). For intracellular Foxp3, Ki67, Eomes and YFP detection, fixation and permeabilization were done according to the manufacturer’s guidelines (Foxp3 transcription factor buffer set, eBioscience) and cells stained with anti-Foxp3, anti-Ki67, anti-Eomes (eBioscience), and anti-GFP rabbit polyclonal antibody (Life Technologies, Carlsbad, CA). For intracellular cytokine detection, spleen cells (3x10^6^) in complete medium were stimulated with LCMV peptides (Research Technologies Brach, Protein Chemistry, NIAID): GP33-41 (GP33, 1 μg/ml), GP 276–286 (GP276, 1 μg/ml), NP396-404 (NP396, 1 μg/ml) and GP 61–80 (GP61, 10 μg/ml) along with GolgiStop (2 mM/ml, BD Biosciences) for 5 hrs at 37 ^o^C. TIL were stimulated with cell stimulation cocktail (eBioscience) containing PMA, ionomycin, Brefeldin A, and monensin for 5 hrs at 37 ^o^C. Later cells were washed, fixed and permeabilized and stained with intracellular cytokine antibodies (anti-IFN-γ and anti-GrB, BD Biosciences, and anti-TNF-α, eBioscience) for overnight at 4 ^o^C. For MHC class I tetramer staining, H-2D^b^ GP33, H-2D^b^ GP276 and H-2D^b^ NP396 (NIH tetramer core facility) were used at 1:100 dilutions and staining was done at 4 ^o^C for 1 hr, and for MHC class II tetramers, IA^b^ GP66-77 (GP66) (NIH tetramer core facility) used at 1:75 dilution and staining performed for 90 mins at 37 ^o^C. Cells were washed and acquired by BD LSRII and LSRFortessa (BD Biosciences) flow cytometers with FASCDiva software.

### Cell sorting

Foxp3^YFP-Cre^ and IFNAR^fl/fl^ x Foxp3^YFP-Cre^ mice (four to five mice per group) were infected with Armstrong and Cl-13 virus. On day 5 post Armstrong and day 25 post Cl-13 infection, spleen and lymph nodes were harvested and single cell suspension were prepared. T cells were isolated by labeling single suspension with CD90.2 microbeads (Miltenyi Biotec, San Diego, CA) and purified through LS columns (Miltenyi Biotec). Purified T cells were stained with anti-CD4 (RM 4–5) for 30 minutes on ice and CD4^+^YFP^+^ cells (5x10^5^/sample) sorted (purity ~95%) by using FACSAria flow cytometer (BD Biosciences).

### Gene expression profiling and bioinformatics analysis

Armstrong (day 5) and chronic LCMV infected (day 25) Foxp3^YFP-Cre^ and IFNAR^fl/fl^xFoxp3^YFP-Cre^ mice CD4^+^YFP^+^ sorted cells were lysed in RLT buffer (Qiagen, Valencia, CA). Total RNA was extracted using Qiagen AllPrep 96 DNA/RNA kit as described by the manufacturer (Qiagen, Valencia, CA), with one exception prior to the extraction, the RLT lysate was homogenized using Qiagen QIAShredder columns (Qiagen) to shear any contaminating gDNA. Samples were then subjected to on-column Dnase I treatment. All steps were performed using PCR amplicon-free laboratory equipment to further minimize background signal during RNA sequencing and library generation. A 150 ng aliquot from each sample was individually adjusted to 50 μl using nuclease-free water. Each sample was processed using Truseq Stranded mRNA Sample Preparation, Rev. E (Illumina Inc., San Diego, CA) using the included barcodes with the following modification: post-amplification libraries were purified with Ampure XP beads twice. The resulting DNA libraries were fragment-sized using a DNA1000 Bioanalyzer Chip (Agilent Technologies, Santa Clara, CA) and quantitated using KAPA Library Quant Kit with universal qPCR Mix (Kapa Biosystems, Wilmington, MA) on a CFX96 Real-Time System (BioRad, Hercules, CA). All eight-ten samples were diluted to a 2 nM working stock and pooled using equal volume amounts. An 11 pM titration point was used to cluster a paired end, RAPID 2-lane flowcell on a Hiseq 2500 DNA sequencer (Illumina). Libraries were run as 2 x 100 bp paired end reads on 2 lanes of an Illumina Hiseq 2500 sequencer, which produced ~28.7 million reads per sample. Reads were trimmed for adapter sequence and filtered for low quality sequence using the FASTX-Toolkit. Remaining reads were mapped to the mouse genome assembly mm10 using Hisat2 [80]. Reads mapping to genes were counted using htseq-count [81]. Differential expression analysis was performed using the Bioconductor package DESeq2 [82]. Further analysis was performed using Partek Genomic Suite (Partek Incorporated) and Ingenuity pathway analysis (IPA) is used for obtaining top canonical pathways, networks based on co-expression, transcription factor binding sites and protein-protein interactions.

### Gene set enrichment analysis

GSEA were performed on the set of 412 genes (Armstrong infection) using GSEA v2.2.3 from The Broad Institute [83]. GSEA was run using molecular signature database v.5.2 gene sets, except the C1: positional gene sets, with 1000 permutations and all default parameters except minimum size of 5.

### Real time quantitative PCR

Total RNA samples from Foxp3^YFP-Cre^ and IFNAR^fl/fl^ x Foxp3^YFP-Cre^ mice were extracted as described in the NGS gene expression profiling method section. cDNAs were prepared from Superscript IV first-strand cDNA synthesis kit (Thermo Fisher Scientific, Waltham, MA) according to the manufacturer’s instructions. Presynthesized Taqman gene expression assays (Thermo Fisher Scientific) were used to amplify *Akap1*, *Car2*, *Cpe*, *Eomes*, *Erdr1*, *Gpat2*, *Rab4a*, *Rell1*, *Rasgrf2*, *Sdc3*, *Tlr7*, and *Actb* was used as internal control. Real time qPCR was performed using QuantStudio7 Flex Real time PCR system (Thermo Fisher Scientific) using Taqman universal master mix II with UNG (Thermo Fisher Scientific). Target gene expressions were calculated by 2^-dct^ and expressed as relative to *Actb*.

### Tumor models

Murine colon adenocarcinoma cells, MC38 cells (ATCC) and melanoma cells, B16.F10 (ATCC) were grown in complete DMEM medium [DMEM/RPMI supplemented with 10% heat-inactivated FBS, L-glutamine (2 mM), sodium pyruvate (1 mM), HEPES (1 mM), non-essential amino acids (0.1 mM), 2-mercaptoethanol (50 μM), and penicillin and streptomycin (100 U/ml)]. Sex- and age-matched IFNAR^fl/fl^ and IFNAR^fl/fl^xFoxp3^YFP-Cre^ mice were injected with 2x10^5^ MC38 cells and or 1.25x10^5^ B16.F10 cells diluted in sterile 1xPBS, subcutaneously (right flank region). Tumor growths were measured in regular intervals by digital calipers (Fisher Scientific), and tumor volumes were calculated by the formulas: length x width x depth. On day 18 post tumor implant, tumors were excised in sterile conditions, and TIL were prepared after the mincing the tumor, and digesting with 1x HBSS containing collagenase type IV (0.5mg/ml), Dnase I (0.1 mg/ml) and Hyaluronidase (2.5 units/ml) for 1 hr at 37 ^o^C. Later, digested cells were washed and treated with ACK lysing buffer to lyse RBCs, and then TIL were purified by density gradient centrifugation using buffered percoll (Sigma-Aldrich, 80%/40%).

### Quantification and statistical analysis

Flow cytometry data were analyzed using FlowJo software version 10.2 and or 10.3 (FlowJo LLC, Ashland, OR). Graphs were prepared by GraphPad Prism software version 7.0 (GraphPad Software, Inc. La Jolla, CA). Statistical analysis was done through unpaired two-tailed Student’s t-test. All data in the graphs presented as Mean±SEM values, and error bars represent SEM. Data were considered statistically significant when *P* < 0.05, and represented as * *P* < 0.05, ** *P* < 0.01, *** *P* < 0.001, and **** *P* < 0.0001.

### Data and software availability

RNA sequence information reported in this study is deposited in NCBI GEO under the accession number: GSE104517.

## Supporting information

S1 Fig Lack of IFNAR signaling in Tregs leads to higher Treg numbers and enhanced activation during LCMV Armstrong infection. (**A**) Representative dot plots demonstrating the deletion of IFNAR on gated CD4^+^Foxp3^+^ Tregs of IFNAR^fl/fl^ x Foxp3^YFP-Cre^ naive mice. (**B**) Spleen cells from naive, D4, D5, D7 and D14 LCMV Armstrong infected IFNAR^fl/fl^ and IFNAR^fl/fl^ x Foxp3^YFP-Cre^ mice were analyzed for CD4^+^Foxp3^+^ Tregs, CD4^+^Foxp3^-^ and CD8^+^ effector T cell frequencies and total numbers. (**C**) Frequencies and absolute numbers of CD4^+^Foxp3^+^CD44^hi^ within CD4^+^Foxp3^+^ T cells of naive, D5, D7 and D14 Armstrong infected IFNAR^fl/fl^ and IFNAR^fl/fl^ x Foxp3^YFP-Cre^ mice. Splenocytes from day 5 LCMV Armstrong infected mice were analyzed for frequencies and absolute numbers of Ki-67^+^ (**D**), ICOS^+^, and TIGIT^+^ cells (**E**) among CD4^+^Foxp3^+^ Tregs. (**F**) Spleen cells from day 7 Armstrong infected mice were analyzed for CD44^hi^ cells within gated CD4^+^Foxp3^-^ T cells and CD8^+^ T cells. * *P* < 0.05, ** *P* < 0.01, *** *P* < 0.001, and **** *P* < 0.0001 (unpaired two-tailed Student’s *t*-test). Data are shown from more than five experiments (**A**), three experiments for naive and two experiments (D4, D5, D7 and D14 infected mice) (**B** and **C)**, and a representative experiment of two experiments (**D, E**, **F**). Each experiment involved groups of four to five mice (Mean±SEM).
(TIF)

S2 Fig CD4^+^ and CD8^+^ effector T cells kinetics and morbidity during chronic LCMV infection. (**A** and **B**) Spleen cells from Day 25 and day 46 Cl-13 infected IFNAR^fl/fl^ and IFNAR^fl/fl^ x Foxp3^YFP-Cre^ mice were analyzed for frequencies and absolute numbers of CD4^+^Foxp3^-^ and CD4^+^Foxp3^-^CD44^hi^ T cells. (**C** and **D**) Spleen cells from Day 25 and day 46 Cl-13 infected IFNAR^fl/fl^ and IFNAR^fl/fl^ x Foxp3^YFP-Cre^ mice were analyzed for frequencies and absolute numbers of CD8^+^ and CD8^+^ CD44^hi^ T cells. (**E**) Body weights were measured on regular intervals, and % change in body weights during chronic LCMV infection were shown. ** *P* < 0.01, ** *P* < 0.01, and *** *P* < 0.001 (unpaired two-tailed Student’s *t*-test). Data are shown from two to four experiments (**A-E**) on indicated days involving six to thirteen mice per group (Mean±SEM).
(TIF)

S3 Fig Absence of IFNAR signaling in Tregs results in a modest increase of virus-specific CD4^+^Foxp3^-^ T cells. (**A**) Spleen cells from chronic LCMV infected (day 25 and day 46) IFNAR^fl/fl^ and IFNAR^fl/fl^ x Foxp3^YFP-Cre^ mice were analyzed for GP66 Tet^+^ within CD4^+^Foxp3^-^CD44^+^ T cells. (**B**) Spleen cells from day 25 and day 46 Cl-13 infected mice were stimulated with GP61. Frequencies and absolute numbers of IFN-γ^+^ and TNF-α^+^ cytokine producing cells within CD4^+^Foxp3^-^CD44^+^GP66 Tet^+^ T cells are shown. * *P* < 0.05 (unpaired two-tailed Student’s *t*-test). Data are shown from one to two representative experiments (**A** and **B**) involving three to eight mice per group (Mean±SEM).
(TIF)

S4 Fig Absence of IFNAR signaling in Tregs results in enhanced virus-specific T cell exhaustion. PD1 expression was evaluated within CD8^+^CD44^+^NP396 Tet^+^ T cells (**A**), and CD4^+^Foxp3^-^CD44^+^GP66 Tet^+^ T cells (**B**) from day 46 Cl-13 infected mice. (**C**) Kinetics of PD1 expression is shown on gated CD4^+^Foxp3^+^ T cells during chronic LCMV infection. * *P* < 0.05 and ** *P* < 0.01 (unpaired two-tailed Student’s *t*-test). Data are shown from a representative of two experiments (**A** and **B**), and from five experiments (**C**) with three to four mice per group in each experiment (Mean±SEM).
(TIF)

S5 Fig Absence of IFNAR signaling on Tregs results in defective generation of memory T cells in chronic LCMV infection. (**A**) Spleen cells from chronic LCMV infected (day 46) IFNAR^fl/fl^ and IFNAR^fl/fl^ x Foxp3^YFP-Cre^ mice were analyzed for frequencies and absolute numbers of GP33 Tet^+^ T cells. Frequencies and absolute numbers of CD62L^+^ (**B**), CD127^+^ (**C**), CXCR3^+^ (**D**), and KLRG1^+^ (**E**) cells were determined within gated CD8^+^CD44^+^GP33 Tet^+^ T cells as in **A**. * *P* < 0.05, ** *P* < 0.01, and *** *P* < 0.001 (unpaired two-tailed Student’s *t*-test). Data obtained from a representative of two experiments (**A-E**) involving four mice per group in each experiment (Mean± SEM).
(TIF)

S6 Fig Transcriptional profile and Active caspase-3 detection in Tregs during acute LCMV infection. (**A**) Day 5 LCMV Armstrong infected Foxp3^YFP-Cre^ and IFNAR^fl/fl^ x Foxp3^YFP-Cre^ mice sorted CD4^+^YFP^+^ Treg cells were analyzed through RNA-seq (4 samples in each group), heat map showing the significant differential expression of 174 IFN-related genes, differentially expressed genes were normalized by z-score (fold change 1.5 and above, adjusted *P* < 0.05). (**B**) Top canonical pathways derived from IPA of differentially expressed non-IFN related genes from Tregs of Foxp3^YFP-Cre^ and IFNAR^fl/fl^xFoxp3^YFP-Cre^ mice during day 5 LCMV Armstrong infection were shown (adjusted p value < 0.1). (**C**) Top two networks were obtained by IPA based on co-expression, transcription factor binding site predictions and protein-protein interactions (genes in green are downregulated, whereas red are upregulated in Foxp3^YFP-Cre^ mice). (**D**) Frequencies and total numbers of CD4^+^Foxp3^+^ Tregs positive for Active Casapse-3 cells are shown from day 5 acute LCMV infected mice. Transcriptome data obtained from an experiment involving four mice per group (**A-C**), and Active caspase-3 detection involved an experiment with four to five mice per group (**D**).
(TIF)

S7 Fig Transcriptional profile analysis and validation of some of the genes in chronic LCMV infection. (**A**) Day 25 LCMV Cl-13 infected Foxp3^YFP-Cre^ and IFNAR^fl/fl^ x Foxp3^YFP-Cre^ mice sorted CD4^+^YFP^+^ Treg cells were analyzed through RNA-seq (5 samples in each group), heat map showing the significant differential expression of 14 IFN-related genes, differentially expressed genes were normalized by z-score (fold change 1.5 and above, adjusted *P* < 0.05). (**B**) Top canonical pathways obtained from IPA of differentially expressed non-IFN related genes from Tregs of Foxp3^YFP-Cre^ and IFNAR^fl/fl^xFoxp3^YFP-Cre^ mice during day 25 LCMV Cl-13 infection were shown (adjusted p value < 0.1). (**C**) Top network is derived by IPA based on co-expression, transcription factor binding site predictions and protein-protein interactions (genes in green are downregulated, whereas red are upregulated in Foxp3^YFP-Cre^ mice). (**D**) Sorted CD4^+^YFP^+^ T cells cDNA samples from LCMV Cl-13 (post day 25) infected Foxp3^YFP-Cre^ and IFNAR^fl/fl^ x Foxp3^YFP-Cre^ mice were subjected to qPCR analysis. Gene expressions of *Akap1*, *Car2*, *Cpe*, *Eomes*, *Erdr1*, *Gpat2*, *Rab4a*, *Rasgrf2*, *Rell1*, *Sdc3*, and *Tlr7* were calculated in relative to the *Actb* expression. * *P* < 0.05, ** *P* < 0.01 and *** *P* < 0.001 (unpaired two-tailed Student’s *t*-test). U.D., undetectable levels. Transcriptome data obtained from an experiment involving five mice per group (**A-C**), and data is a representative of an experiment involving five samples obtained from five mice per group (**D**) (Mean±SEM).
(TIF)

S8 Fig Effector T cells proliferate less in MC38 tumor induced IFNAR^fl/fl^ x Foxp3^YFP-Cre^ mice. (**A**) IFNAR^fl/fl^ and IFNAR^fl/fl^ x Foxp3^YFP-Cre^ mice were injected with 2x10^5^ MC38 cells subcutaneously. On day 18 post tumor implantation TIL were isolated, CD4^+^Foxp3^+^ T cell frequencies were ascertained. (**B** and **C**) Ki67 expression by tumor infiltrating CD4^+^Foxp3^-^ and CD8^+^ T cells was evaluated. * *P* < 0.05 (unpaired two-tailed Student’s *t*-test). Data shown from two experiments (**A**), and from a representative of two independent experiments (**B** and **C**) involving three to five mice per group in each experiment (Mean±SEM).
(TIF)

S1 Table Differential expression of genes associated with cell cycle, DNA replication, recombination, repair and cancer. (XLSX)

S2 Table Differential expression of genes associated with cellular movement, hematological system development and function, immune cell trafficking. (XLSX)

## References

[1] Fontenot JD, Rudensky AY. A well adapted regulatory contrivance: regulatory T cell development and the forkhead family transcription factor Foxp3. Nature immunology. 2005;6(4):331–7. doi: 10.1038/ni1179 . 15785758 10.1038/ni1179

[2] Sakaguchi S. Naturally arising CD4+ regulatory t cells for immunologic self-tolerance and negative control of immune responses. Annu Rev Immunol. 2004;22:531–62. doi: 10.1146/annurev.immunol.21.120601.141122 . 15032588 10.1146/annurev.immunol.21.120601.141122

[3] Shevach EM. Mechanisms of foxp3+ T regulatory cell-mediated suppression. Immunity. 2009;30(5):636–45. doi: 10.1016/j.immuni.2009.04.010 . 19464986 10.1016/j.immuni.2009.04.010

[4] Belkaid Y, Blank RB, Suffia I. Natural regulatory T cells and parasites: a common quest for host homeostasis. Immunol Rev. 2006;212:287–300. doi: 10.1111/j.0105-2896.2006.00409.x . 16903921 10.1111/j.0105-2896.2006.00409.x

[5] Belkaid Y, Piccirillo CA, Mendez S, Shevach EM, Sacks DL. CD4+CD25+ regulatory T cells control Leishmania major persistence and immunity. Nature. 2002;420(6915):502–7. doi: 10.1038/nature01152 . 12466842 10.1038/nature01152

[6] Facciabene A, Motz GT, Coukos G. T-regulatory cells: key players in tumor immune escape and angiogenesis. Cancer Res. 2012;72(9):2162–71. doi: 10.1158/0008-5472.CAN-11-3687 ; PubMed Central PMCID: PMCPMC3342842. 22549946 10.1158/0008-5472.CAN-11-3687 PMC3342842

[7] Johanns TM, Ertelt JM, Rowe JH, Way SS. Regulatory T cell suppressive potency dictates the balance between bacterial proliferation and clearance during persistent Salmonella infection. PLoS pathogens. 2010;6(8):e1001043 doi: 10.1371/journal.ppat.1001043 ; PubMed Central PMCID: PMCPMC2920851. 20714351 10.1371/journal.ppat.1001043 PMC2920851

[8] Rowe JH, Ertelt JM, Aguilera MN, Farrar MA, Way SS. Foxp3(+) regulatory T cell expansion required for sustaining pregnancy compromises host defense against prenatal bacterial pathogens. Cell Host Microbe. 2011;10(1):54–64. doi: 10.1016/j.chom.2011.06.005 ; PubMed Central PMCID: PMCPMC3140139. 21767812 10.1016/j.chom.2011.06.005 PMC3140139

[9] Scott-Browne JP, Shafiani S, Tucker-Heard G, Ishida-Tsubota K, Fontenot JD, Rudensky AY, et al Expansion and function of Foxp3-expressing T regulatory cells during tuberculosis. The Journal of experimental medicine. 2007;204(9):2159–69. doi: 10.1084/jem.20062105 ; PubMed Central PMCID: PMCPMC2118702. 17709423 10.1084/jem.20062105 PMC2118702

[10] Cabrera R, Tu Z, Xu Y, Firpi RJ, Rosen HR, Liu C, et al An immunomodulatory role for CD4(+)CD25(+) regulatory T lymphocytes in hepatitis C virus infection. Hepatology. 2004;40(5):1062–71. doi: 10.1002/hep.20454 . 15486925 10.1002/hep.20454

[11] Luhn K, Simmons CP, Moran E, Dung NT, Chau TN, Quyen NT, et al Increased frequencies of CD4+ CD25(high) regulatory T cells in acute dengue infection. The Journal of experimental medicine. 2007;204(5):979–85. doi: 10.1084/jem.20061381 ; PubMed Central PMCID: PMCPMC2118571. 17452519 10.1084/jem.20061381 PMC2118571

[12] Lanteri MC, O'Brien KM, Purtha WE, Cameron MJ, Lund JM, Owen RE, et al Tregs control the development of symptomatic West Nile virus infection in humans and mice. J Clin Invest. 2009;119(11):3266–77. doi: 10.1172/JCI39387 ; PubMed Central PMCID: PMCPMC2769173. 19855131 10.1172/JCI39387 PMC2769173

[13] Lund JM, Hsing L, Pham TT, Rudensky AY. Coordination of early protective immunity to viral infection by regulatory T cells. Science. 2008;320(5880):1220–4. doi: 10.1126/science.1155209 ; PubMed Central PMCID: PMCPMC2519146. 18436744 10.1126/science.1155209 PMC2519146

[14] Moreno-Fernandez ME, Rueda CM, Rusie LK, Chougnet CA. Regulatory T cells control HIV replication in activated T cells through a cAMP-dependent mechanism. Blood. 2011;117(20):5372–80. doi: 10.1182/blood-2010-12-323162 ; PubMed Central PMCID: PMCPMC3109711. 21436067 10.1182/blood-2010-12-323162 PMC3109711

[15] Hori S, Carvalho TL, Demengeot J. CD25+CD4+ regulatory T cells suppress CD4+ T cell-mediated pulmonary hyperinflammation driven by Pneumocystis carinii in immunodeficient mice. European journal of immunology. 2002;32(5):1282–91. doi: 10.1002/1521-4141(200205)32:5&lt;1282::AID-IMMU1282&gt;3.0.CO;2-# . 11981815 10.1002/1521-4141(200205)32:5<1282::AID-IMMU1282>3.0.CO;2-#

[16] Layland LE, Straubinger K, Ritter M, Loffredo-Verde E, Garn H, Sparwasser T, et al Schistosoma mansoni-mediated suppression of allergic airway inflammation requires patency and Foxp3+ Treg cells. PLoS Negl Trop Dis. 2013;7(8):e2379 doi: 10.1371/journal.pntd.0002379 ; PubMed Central PMCID: PMCPMC3744427. 23967364 10.1371/journal.pntd.0002379 PMC3744427

[17] Haque A, Best SE, Amante FH, Mustafah S, Desbarrieres L, de Labastida F, et al CD4+ natural regulatory T cells prevent experimental cerebral malaria via CTLA-4 when expanded in vivo. PLoS pathogens. 2010;6(12):e1001221 doi: 10.1371/journal.ppat.1001221 ; PubMed Central PMCID: PMCPMC3000360. 21170302 10.1371/journal.ppat.1001221 PMC3000360

[18] Oldenhove G, Bouladoux N, Wohlfert EA, Hall JA, Chou D, Dos Santos L, et al Decrease of Foxp3+ Treg cell number and acquisition of effector cell phenotype during lethal infection. Immunity. 2009;31(5):772–86. doi: 10.1016/j.immuni.2009.10.001 ; PubMed Central PMCID: PMCPMC2814877. 19896394 10.1016/j.immuni.2009.10.001 PMC2814877

[19] Pandiyan P, Conti HR, Zheng L, Peterson AC, Mathern DR, Hernandez-Santos N, et al CD4(+)CD25(+)Foxp3(+) regulatory T cells promote Th17 cells in vitro and enhance host resistance in mouse Candida albicans Th17 cell infection model. Immunity. 2011;34(3):422–34. doi: 10.1016/j.immuni.2011.03.002 ; PubMed Central PMCID: PMCPMC3258585. 21435589 10.1016/j.immuni.2011.03.002 PMC3258585

[20] Lewerenz M, Mogensen KE, Uze G. Shared receptor components but distinct complexes for alpha and beta interferons. Journal of molecular biology. 1998;282(3):585–99. doi: 10.1006/jmbi.1998.2026 . 9737924 10.1006/jmbi.1998.2026

[21] Ivashkiv LB, Donlin LT. Regulation of type I interferon responses. Nature reviews Immunology. 2014;14(1):36–49. doi: 10.1038/nri3581 ; PubMed Central PMCID: PMC4084561. 24362405 10.1038/nri3581 PMC4084561

[22] Chow KT, Gale M Jr. SnapShot: Interferon Signaling. Cell. 2015;163(7):1808–e1. doi: 10.1016/j.cell.2015.12.008 . 26687364 10.1016/j.cell.2015.12.008

[23] Yan N, Chen ZJ. Intrinsic antiviral immunity. Nature immunology. 2012;13(3):214–22. doi: 10.1038/ni.2229 ; PubMed Central PMCID: PMC3549670. 22344284 10.1038/ni.2229 PMC3549670

[24] Al Moussawi K, Ghigo E, Kalinke U, Alexopoulou L, Mege JL, Desnues B. Type I interferon induction is detrimental during infection with the Whipple's disease bacterium, Tropheryma whipplei. PLoS pathogens. 2010;6(1):e1000722 doi: 10.1371/journal.ppat.1000722 ; PubMed Central PMCID: PMC2798751. 20090833 10.1371/journal.ppat.1000722 PMC2798751

[25] Fink K, Lang KS, Manjarrez-Orduno N, Junt T, Senn BM, Holdener M, et al Early type I interferon-mediated signals on B cells specifically enhance antiviral humoral responses. European journal of immunology. 2006;36(8):2094–105. doi: 10.1002/eji.200635993 . 16810635 10.1002/eji.200635993

[26] Havenar-Daughton C, Kolumam GA, Murali-Krishna K. Cutting Edge: The direct action of type I IFN on CD4 T cells is critical for sustaining clonal expansion in response to a viral but not a bacterial infection. Journal of immunology. 2006;176(6):3315–9. . 16517698 10.4049/jimmunol.176.6.3315

[27] Kolumam GA, Thomas S, Thompson LJ, Sprent J, Murali-Krishna K. Type I interferons act directly on CD8 T cells to allow clonal expansion and memory formation in response to viral infection. The Journal of experimental medicine. 2005;202(5):637–50. doi: 10.1084/jem.20050821 ; PubMed Central PMCID: PMC2212878. 16129706 10.1084/jem.20050821 PMC2212878

[28] McNab F, Mayer-Barber K, Sher A, Wack A, O'Garra A. Type I interferons in infectious disease. Nature reviews Immunology. 2015;15(2):87–103. doi: 10.1038/nri3787 . 25614319 10.1038/nri3787 PMC7162685

[29] Nguyen KB, Salazar-Mather TP, Dalod MY, Van Deusen JB, Wei XQ, Liew FY, et al Coordinated and distinct roles for IFN-alpha beta, IL-12, and IL-15 regulation of NK cell responses to viral infection. Journal of immunology. 2002;169(8):4279–87. . 12370359 10.4049/jimmunol.169.8.4279

[30] Pace L, Vitale S, Dettori B, Palombi C, La Sorsa V, Belardelli F, et al APC activation by IFN-alpha decreases regulatory T cell and enhances Th cell functions. Journal of immunology. 2010;184(11):5969–79. doi: 10.4049/jimmunol.0900526 . 20427775 10.4049/jimmunol.0900526

[31] Snell LM, McGaha TL, Brooks DG. Type I Interferon in Chronic Virus Infection and Cancer. Trends Immunol. 2017;38(8):542–57. doi: 10.1016/j.it.2017.05.005 . 28579323 10.1016/j.it.2017.05.005 PMC8059441

[32] Stifter SA, Feng CG. Interfering with immunity: detrimental role of type I IFNs during infection. Journal of immunology. 2015;194(6):2455–65. doi: 10.4049/jimmunol.1402794 . 25747907 10.4049/jimmunol.1402794

[33] Wilson EB, Yamada DH, Elsaesser H, Herskovitz J, Deng J, Cheng G, et al Blockade of chronic type I interferon signaling to control persistent LCMV infection. Science. 2013;340(6129):202–7. doi: 10.1126/science.1235208 ; PubMed Central PMCID: PMCPMC3704950. 23580528 10.1126/science.1235208 PMC3704950

[34] Teijaro JR, Ng C, Lee AM, Sullivan BM, Sheehan KC, Welch M, et al Persistent LCMV infection is controlled by blockade of type I interferon signaling. Science. 2013;340(6129):207–11. doi: 10.1126/science.1235214 ; PubMed Central PMCID: PMCPMC3640797. 23580529 10.1126/science.1235214 PMC3640797

[35] Namdar A, Nikbin B, Ghabaee M, Bayati A, Izad M. Effect of IFN-beta therapy on the frequency and function of CD4(+)CD25(+) regulatory T cells and Foxp3 gene expression in relapsing-remitting multiple sclerosis (RRMS): a preliminary study. Journal of neuroimmunology. 2010;218(1–2):120–4. doi: 10.1016/j.jneuroim.2009.10.013 . 19932513 10.1016/j.jneuroim.2009.10.013

[36] Riley CH, Jensen MK, Brimnes MK, Hasselbalch HC, Bjerrum OW, Straten PT, et al Increase in circulating CD4(+)CD25(+)Foxp3(+) T cells in patients with Philadelphia-negative chronic myeloproliferative neoplasms during treatment with IFN-alpha. Blood. 2011;118(8):2170–3. doi: 10.1182/blood-2011-03-340992 . 21708889 10.1182/blood-2011-03-340992

[37] Metidji A, Rieder SA, Glass DD, Cremer I, Punkosdy GA, Shevach EM. IFN-alpha/beta receptor signaling promotes regulatory T cell development and function under stress conditions. Journal of immunology. 2015;194(9):4265–76. doi: 10.4049/jimmunol.1500036 ; PubMed Central PMCID: PMC4402260. 25795758 10.4049/jimmunol.1500036 PMC4402260

[38] Ou R, Zhou S, Huang L, Moskophidis D. Critical role for alpha/beta and gamma interferons in persistence of lymphocytic choriomeningitis virus by clonal exhaustion of cytotoxic T cells. J Virol. 2001;75(18):8407–23. doi: 10.1128/JVI.75.18.8407-8423.2001 ; PubMed Central PMCID: PMCPMC115086. 11507186 10.1128/JVI.75.18.8407-8423.2001 PMC115086

[39] Srivastava S, Koch MA, Pepper M, Campbell DJ. Type I interferons directly inhibit regulatory T cells to allow optimal antiviral T cell responses during acute LCMV infection. The Journal of experimental medicine. 2014;211(5):961–74. doi: 10.1084/jem.20131556 ; PubMed Central PMCID: PMC4010906. 24711580 10.1084/jem.20131556 PMC4010906

[40] Bennett CL, Christie J, Ramsdell F, Brunkow ME, Ferguson PJ, Whitesell L, et al The immune dysregulation, polyendocrinopathy, enteropathy, X-linked syndrome (IPEX) is caused by mutations of FOXP3. Nat Genet. 2001;27(1):20–1. doi: 10.1038/83713 . 11137993 10.1038/83713

[41] Fontenot JD, Gavin MA, Rudensky AY. Foxp3 programs the development and function of CD4+CD25+ regulatory T cells. Nature immunology. 2003;4(4):330–6. doi: 10.1038/ni904 . 12612578 10.1038/ni904

[42] Penaloza-MacMaster P, Kamphorst AO, Wieland A, Araki K, Iyer SS, West EE, et al Interplay between regulatory T cells and PD-1 in modulating T cell exhaustion and viral control during chronic LCMV infection. The Journal of experimental medicine. 2014;211(9):1905–18. doi: 10.1084/jem.20132577 ; PubMed Central PMCID: PMCPMC4144726. 25113973 10.1084/jem.20132577 PMC4144726

[43] Buggert M, Tauriainen J, Yamamoto T, Frederiksen J, Ivarsson MA, Michaelsson J, et al T-bet and Eomes are differentially linked to the exhausted phenotype of CD8+ T cells in HIV infection. PLoS pathogens. 2014;10(7):e1004251 doi: 10.1371/journal.ppat.1004251 ; PubMed Central PMCID: PMCPMC4102564. 25032686 10.1371/journal.ppat.1004251 PMC4102564

[44] Gupta PK, Godec J, Wolski D, Adland E, Yates K, Pauken KE, et al CD39 Expression Identifies Terminally Exhausted CD8+ T Cells. PLoS pathogens. 2015;11(10):e1005177 doi: 10.1371/journal.ppat.1005177 ; PubMed Central PMCID: PMCPMC4618999. 26485519 10.1371/journal.ppat.1005177 PMC4618999

[45] Park HJ, Park JS, Jeong YH, Son J, Ban YH, Lee BH, et al PD-1 upregulated on regulatory T cells during chronic virus infection enhances the suppression of CD8+ T cell immune response via the interaction with PD-L1 expressed on CD8+ T cells. Journal of immunology. 2015;194(12):5801–11. doi: 10.4049/jimmunol.1401936 . 25934860 10.4049/jimmunol.1401936

[46] Wherry EJ, Kurachi M. Molecular and cellular insights into T cell exhaustion. Nature reviews Immunology. 2015;15(8):486–99. doi: 10.1038/nri3862 ; PubMed Central PMCID: PMCPMC4889009. 26205583 10.1038/nri3862 PMC4889009

[47] Laidlaw BJ, Cui W, Amezquita RA, Gray SM, Guan T, Lu Y, et al Production of IL-10 by CD4(+) regulatory T cells during the resolution of infection promotes the maturation of memory CD8(+) T cells. Nature immunology. 2015;16(8):871–9. doi: 10.1038/ni.3224 ; PubMed Central PMCID: PMCPMC4713030. 26147684 10.1038/ni.3224 PMC4713030

[48] Rusinova I, Forster S, Yu S, Kannan A, Masse M, Cumming H, et al Interferome v2.0: an updated database of annotated interferon-regulated genes. Nucleic Acids Res. 2013;41(Database issue):D1040–6. doi: 10.1093/nar/gks1215 ; PubMed Central PMCID: PMCPMC3531205. 23203888 10.1093/nar/gks1215 PMC3531205

[49] Fu W, Ergun A, Lu T, Hill JA, Haxhinasto S, Fassett MS, et al A multiply redundant genetic switch 'locks in' the transcriptional signature of regulatory T cells. Nature immunology. 2012;13(10):972–80. doi: 10.1038/ni.2420 ; PubMed Central PMCID: PMCPMC3698954. 22961053 10.1038/ni.2420 PMC3698954

[50] van der Veeken J, Gonzalez AJ, Cho H, Arvey A, Hemmers S, Leslie CS, et al Memory of Inflammation in Regulatory T Cells. Cell. 2016;166(4):977–90. doi: 10.1016/j.cell.2016.07.006 ; PubMed Central PMCID: PMCPMC4996371. 27499023 10.1016/j.cell.2016.07.006 PMC4996371

[51] Pidoux G, Gerbaud P, Dompierre J, Lygren B, Solstad T, Evain-Brion D, et al A PKA-ezrin-Cx43 signaling complex controls gap junction communication and thereby trophoblast cell fusion. J Cell Sci. 2014;127(Pt 19):4172–85. doi: 10.1242/jcs.149609 . 25052094 10.1242/jcs.149609

[52] Wehbi VL, Tasken K. Molecular Mechanisms for cAMP-Mediated Immunoregulation in T cells—Role of Anchored Protein Kinase A Signaling Units. Front Immunol. 2016;7:222 doi: 10.3389/fimmu.2016.00222 ; PubMed Central PMCID: PMCPMC4896925. 27375620 10.3389/fimmu.2016.00222 PMC4896925

[53] Ishiguro K, Green T, Rapley J, Wachtel H, Giallourakis C, Landry A, et al Ca2+/calmodulin-dependent protein kinase II is a modulator of CARMA1-mediated NF-kappaB activation. Mol Cell Biol. 2006;26(14):5497–508. doi: 10.1128/MCB.02469-05 ; PubMed Central PMCID: PMCPMC1592706. 16809782 10.1128/MCB.02469-05 PMC1592706

[54] Long M, Park SG, Strickland I, Hayden MS, Ghosh S. Nuclear factor-kappaB modulates regulatory T cell development by directly regulating expression of Foxp3 transcription factor. Immunity. 2009;31(6):921–31. doi: 10.1016/j.immuni.2009.09.022 . 20064449 10.1016/j.immuni.2009.09.022

[55] Konig S, Probst-Kepper M, Reinl T, Jeron A, Huehn J, Schraven B, et al First insight into the kinome of human regulatory T cells. PLoS One. 2012;7(7):e40896 doi: 10.1371/journal.pone.0040896 ; PubMed Central PMCID: PMCPMC3397934. 22815858 10.1371/journal.pone.0040896 PMC3397934

[56] Oaks Z, Winans T, Huang N, Blair S, Beckford M, Banki K, et al., editors. Rab4A Is Required for Development of Tregs, Restricts Antiphospholipid Antibody Production and Pro-Inflammatory Expansion of Macrophages and Neutrophils, and Blocks Pristane-Induced Intra-Alveolar Hemorrhage in a Mouse Model of SLE. Arthritis Rheumatol; 2016 Sep. ACR/ARHP Annual Meeting, 2016.

[57] Ruiz S, Santos E, Bustelo XR. RasGRF2, a guanosine nucleotide exchange factor for Ras GTPases, participates in T-cell signaling responses. Mol Cell Biol. 2007;27(23):8127–42. doi: 10.1128/MCB.00912-07 ; PubMed Central PMCID: PMCPMC2169177. 17923690 10.1128/MCB.00912-07 PMC2169177

[58] Schmitz I, Schneider C, Frohlich A, Frebel H, Christ D, Leonard WJ, et al IL-21 Restricts Virus-driven Treg Cell Expansion in Chronic LCMV Infection. PLoS pathogens. 2013;9(5):e1003362 doi: 10.1371/journal.ppat.1003362 23696736 10.1371/journal.ppat.1003362 PMC3656089

[59] Cusick JK, Mustian A, Goldberg K, Reyland ME. RELT induces cellular death in HEK 293 epithelial cells. Cell Immunol. 2010;261(1):1–8. doi: 10.1016/j.cellimm.2009.10.013 ; PubMed Central PMCID: PMCPMC3407663. 19969290 10.1016/j.cellimm.2009.10.013 PMC3407663

[60] Hackl D, Loschko J, Sparwasser T, Reindl W, Krug AB. Activation of dendritic cells via TLR7 reduces Foxp3 expression and suppressive function in induced Tregs. European journal of immunology. 2011;41(5):1334–43. doi: 10.1002/eji.201041014 . 21469103 10.1002/eji.201041014

[61] Lee J, Jung MK, Park HJ, Kim KE, Cho D. Erdr1 Suppresses Murine Melanoma Growth via Regulation of Apoptosis. Int J Mol Sci. 2016;17(1). doi: 10.3390/ijms17010107 ; PubMed Central PMCID: PMCPMC4730348. 26784177 10.3390/ijms17010107 PMC4730348

[62] Di Franco S, Turdo A, Todaro M, Stassi G. Role of Type I and II Interferons in Colorectal Cancer and Melanoma. Front Immunol. 2017;8:878 doi: 10.3389/fimmu.2017.00878 ; PubMed Central PMCID: PMCPMC5526853. 28798748 10.3389/fimmu.2017.00878 PMC5526853

[63] Stewart CA, Metheny H, Iida N, Smith L, Hanson M, Steinhagen F, et al Interferon-dependent IL-10 production by Tregs limits tumor Th17 inflammation. J Clin Invest. 2013;123(11):4859–74. doi: 10.1172/JCI65180 ; PubMed Central PMCID: PMCPMC3809773. 24216477 10.1172/JCI65180 PMC3809773

[64] Benson A, Murray S, Divakar P, Burnaevskiy N, Pifer R, Forman J, et al Microbial infection-induced expansion of effector T cells overcomes the suppressive effects of regulatory T cells via an IL-2 deprivation mechanism. Journal of immunology. 2012;188(2):800–10. doi: 10.4049/jimmunol.1100769 ; PubMed Central PMCID: PMCPMC3253229. 22147768 10.4049/jimmunol.1100769 PMC3253229

[65] Korn T, Reddy J, Gao W, Bettelli E, Awasthi A, Petersen TR, et al Myelin-specific regulatory T cells accumulate in the CNS but fail to control autoimmune inflammation. Nat Med. 2007;13(4):423–31. doi: 10.1038/nm1564 ; PubMed Central PMCID: PMCPMC3427780. 17384649 10.1038/nm1564 PMC3427780

[66] Clough LE, Wang CJ, Schmidt EM, Booth G, Hou TZ, Ryan GA, et al Release from regulatory T cell-mediated suppression during the onset of tissue-specific autoimmunity is associated with elevated IL-21. Journal of immunology. 2008;180(8):5393–401. . 18390721 10.4049/jimmunol.180.8.5393

[67] Wehrens EJ, Vastert SJ, Mijnheer G, Meerding J, Klein M, Wulffraat NM, et al Anti-tumor necrosis factor alpha targets protein kinase B/c-Akt-induced resistance of effector cells to suppression in juvenile idiopathic arthritis. Arthritis Rheum. 2013;65(12):3279–84. doi: 10.1002/art.38132 . 23983021 10.1002/art.38132

[68] Trinschek B, Luessi F, Haas J, Wildemann B, Zipp F, Wiendl H, et al Kinetics of IL-6 production defines T effector cell responsiveness to regulatory T cells in multiple sclerosis. PLoS One. 2013;8(10):e77634 doi: 10.1371/journal.pone.0077634 ; PubMed Central PMCID: PMCPMC3796502. 24155968 10.1371/journal.pone.0077634 PMC3796502

[69] van Amelsfort JM, van Roon JA, Noordegraaf M, Jacobs KM, Bijlsma JW, Lafeber FP, et al Proinflammatory mediator-induced reversal of CD4+,CD25+ regulatory T cell-mediated suppression in rheumatoid arthritis. Arthritis Rheum. 2007;56(3):732–42. doi: 10.1002/art.22414 . 17328044 10.1002/art.22414

[70] O'Sullivan BJ, Thomas HE, Pai S, Santamaria P, Iwakura Y, Steptoe RJ, et al IL-1 beta breaks tolerance through expansion of CD25+ effector T cells. Journal of immunology. 2006;176(12):7278–87. . 16751371 10.4049/jimmunol.176.12.7278

[71] Pace L, Rizzo S, Palombi C, Brombacher F, Doria G. Cutting edge: IL-4-induced protection of CD4+CD25- Th cells from CD4+CD25+ regulatory T cell-mediated suppression. Journal of immunology. 2006;176(7):3900–4. . 16547222 10.4049/jimmunol.176.7.3900

[72] Mercadante ER, Lorenz UM. Breaking Free of Control: How Conventional T Cells Overcome Regulatory T Cell Suppression. Front Immunol. 2016;7:193 doi: 10.3389/fimmu.2016.00193 ; PubMed Central PMCID: PMCPMC4870238. 27242798 10.3389/fimmu.2016.00193 PMC4870238

[73] Stephens GL, McHugh RS, Whitters MJ, Young DA, Luxenberg D, Carreno BM, et al Engagement of glucocorticoid-induced TNFR family-related receptor on effector T cells by its ligand mediates resistance to suppression by CD4+CD25+ T cells. Journal of immunology. 2004;173(8):5008–20. . 15470044 10.4049/jimmunol.173.8.5008

[74] Choi BK, Bae JS, Choi EM, Kang WJ, Sakaguchi S, Vinay DS, et al 4-1BB-dependent inhibition of immunosuppression by activated CD4+CD25+ T cells. J Leukoc Biol. 2004;75(5):785–91. doi: 10.1189/jlb.1003491 . 14694186 10.1189/jlb.1003491

[75] Ishii N, Takahashi T, Soroosh P, Sugamura K. OX40-OX40 ligand interaction in T-cell-mediated immunity and immunopathology. Adv Immunol. 2010;105:63–98. doi: 10.1016/S0065-2776(10)05003-0 . 20510730 10.1016/S0065-2776(10)05003-0

[76] Chen X, Hamano R, Subleski JJ, Hurwitz AA, Howard OM, Oppenheim JJ. Expression of costimulatory TNFR2 induces resistance of CD4+FoxP3- conventional T cells to suppression by CD4+FoxP3+ regulatory T cells. Journal of immunology. 2010;185(1):174–82. doi: 10.4049/jimmunol.0903548 . 20525892 10.4049/jimmunol.0903548 PMC6314668

[77] Nie H, Zheng Y, Li R, Guo TB, He D, Fang L, et al Phosphorylation of FOXP3 controls regulatory T cell function and is inhibited by TNF-alpha in rheumatoid arthritis. Nat Med. 2013;19(3):322–8. doi: 10.1038/nm.3085 . 23396208 10.1038/nm.3085

[78] Ahmed R, Salmi A, Butler LD, Chiller JM, Oldstone MB. Selection of genetic variants of lymphocytic choriomeningitis virus in spleens of persistently infected mice. Role in suppression of cytotoxic T lymphocyte response and viral persistence. The Journal of experimental medicine. 1984;160(2):521–40. ; PubMed Central PMCID: PMCPMC2187458. 6332167 10.1084/jem.160.2.521 PMC2187458

[79] Sebastian M, Lopez-Ocasio M, Metidji A, Rieder SA, Shevach EM, Thornton AM. Helios Controls a Limited Subset of Regulatory T Cell Functions. Journal of immunology. 2016;196(1):144–55. doi: 10.4049/jimmunol.1501704 ; PubMed Central PMCID: PMCPMC4685018. 26582951 10.4049/jimmunol.1501704 PMC4685018

[80] Kim D, Langmead B, Salzberg SL. HISAT: a fast spliced aligner with low memory requirements. Nat Methods. 2015;12(4):357–60. doi: 10.1038/nmeth.3317 ; PubMed Central PMCID: PMCPMC4655817. 25751142 10.1038/nmeth.3317 PMC4655817

[81] Anders S, Pyl PT, Huber W. HTSeq—a Python framework to work with high-throughput sequencing data. Bioinformatics. 2015;31(2):166–9. doi: 10.1093/bioinformatics/btu638 ; PubMed Central PMCID: PMCPMC4287950. 25260700 10.1093/bioinformatics/btu638 PMC4287950

[82] Love MI, Huber W, Anders S. Moderated estimation of fold change and dispersion for RNA-seq data with DESeq2. Genome Biol. 2014;15(12):550 doi: 10.1186/s13059-014-0550-8 ; PubMed Central PMCID: PMCPMC4302049. 25516281 10.1186/s13059-014-0550-8 PMC4302049

[83] Subramanian A, Tamayo P, Mootha VK, Mukherjee S, Ebert BL, Gillette MA, et al Gene set enrichment analysis: a knowledge-based approach for interpreting genome-wide expression profiles. Proc Natl Acad Sci U S A. 2005;102(43):15545–50. doi: 10.1073/pnas.0506580102 ; PubMed Central PMCID: PMCPMC1239896. 16199517 10.1073/pnas.0506580102 PMC1239896

