# Supplementary figures and images for: Type I interferon signaling attenuates regulatory T cell function in viral infection and in the tumor microenvironment
Source: PLoS Pathog. 2018 Apr 19;14(4):e1006985. doi: 10.1371/journal.ppat.1006985 (PMC5929570; doi:10.1371/journal.ppat.1006985)

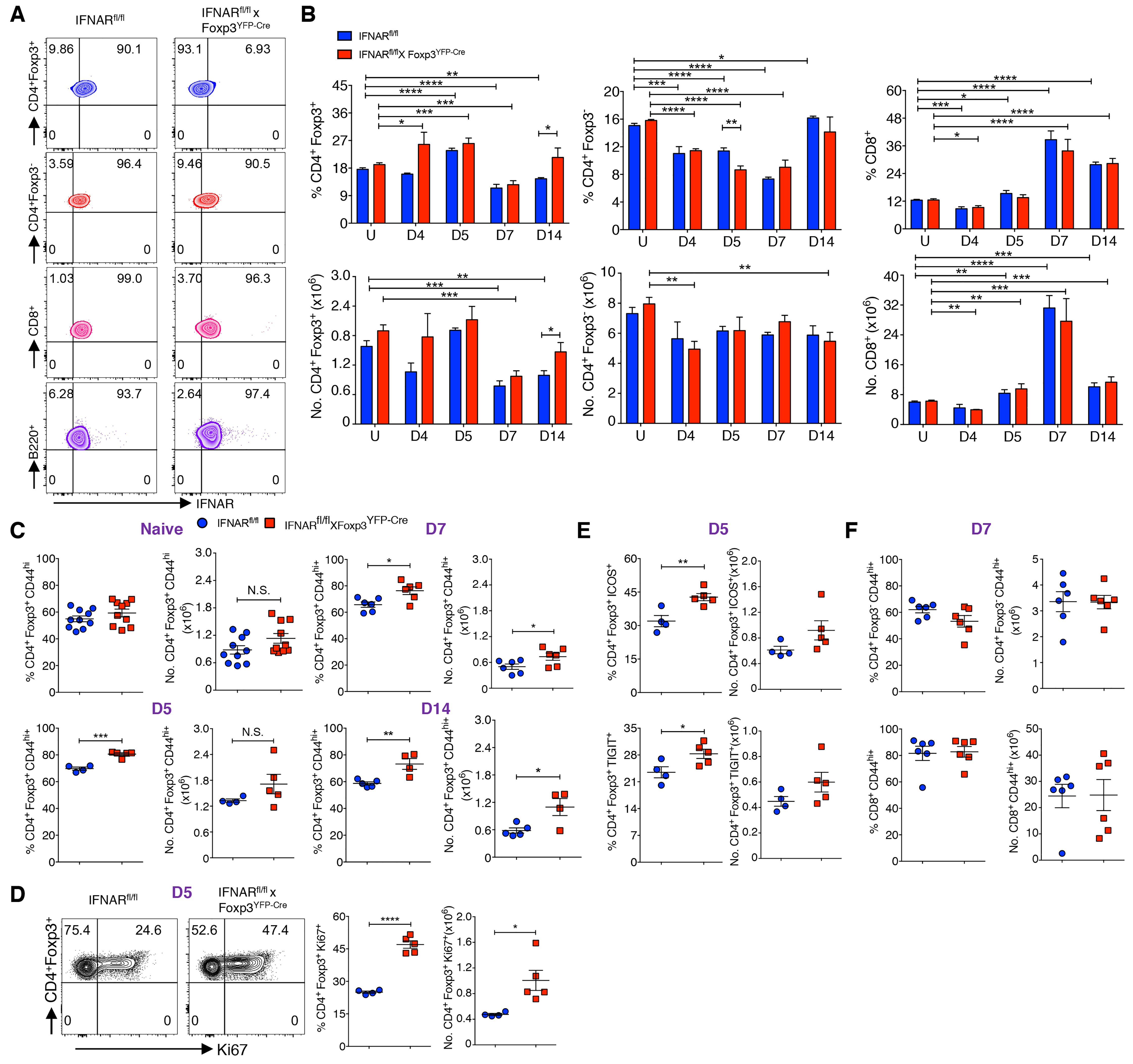

Supplement: S1 Fig — (A) Representative dot plots demonstrating the deletion of IFNAR on gated CD4+Foxp3+ Tregs of IFNARfl/fl x Foxp3YFP-Cre naive mice. (B) Spleen cells from naive, D4, D5, D7 and D14 LCMV Armstrong infected IFNARfl/fl and IFNARfl/fl x Foxp3YFP-Cre mice were analyzed for CD4+Foxp3+ Tregs, CD4+Foxp3- and CD8+ effector T cell frequencies and total numbers. (C) Frequencies and absolute numbers of CD4+Foxp3+CD44hi within CD4+Foxp3+ T cells of naive, D5, D7 and D14 Armstrong infected IFNARfl/fl and IFNARfl/fl x Foxp3YFP-Cre mice. Splenocytes from day 5 LCMV Armstrong infected mice were analyzed for frequencies and absolute numbers of Ki-67+ (D), ICOS+, and TIGIT+ cells (E) among CD4+Foxp3+ Tregs. (F) Spleen cells from day 7 Armstrong infected mice were analyzed for CD44hi cells within gated CD4+Foxp3- T cells and CD8+ T cells. * P < 0.05, ** P < 0.01, *** P < 0.001, and **** P < 0.0001 (unpaired two-tailed Student’s t-test). Data are shown from more than five experiments (A), three experiments for naive and two experiments (D4, D5, D7 and D14 infected mice) (B and C), and a representative experiment of two experiments (D, E, F). Each experiment involved groups of four to five mice (Mean±SEM). (TIF) [file ppat.1006985.s001.tif]

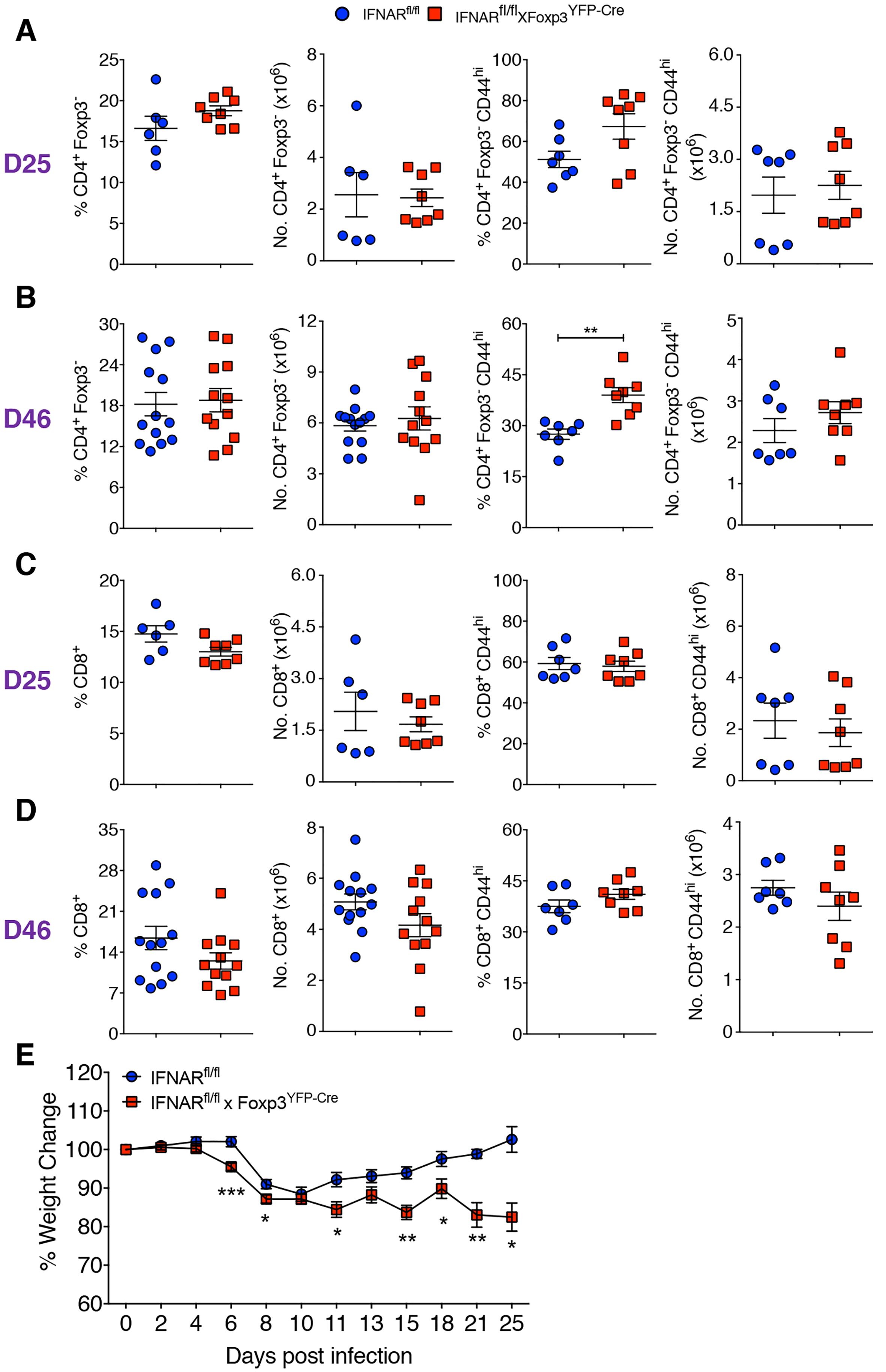

Supplement: S2 Fig — (A and B) Spleen cells from Day 25 and day 46 Cl-13 infected IFNARfl/fl and IFNARfl/fl x Foxp3YFP-Cre mice were analyzed for frequencies and absolute numbers of CD4+Foxp3- and CD4+Foxp3-CD44hi T cells. (C and D) Spleen cells from Day 25 and day 46 Cl-13 infected IFNARfl/fl and IFNARfl/fl x Foxp3YFP-Cre mice were analyzed for frequencies and absolute numbers of CD8+ and CD8+ CD44hi T cells. (E) Body weights were measured on regular intervals, and % change in body weights during chronic LCMV infection were shown. ** P < 0.01, ** P < 0.01, and *** P < 0.001 (unpaired two-tailed Student’s t-test). Data are shown from two to four experiments (A-E) on indicated days involving six to thirteen mice per group (Mean±SEM). (TIF) [file ppat.1006985.s002.tif]

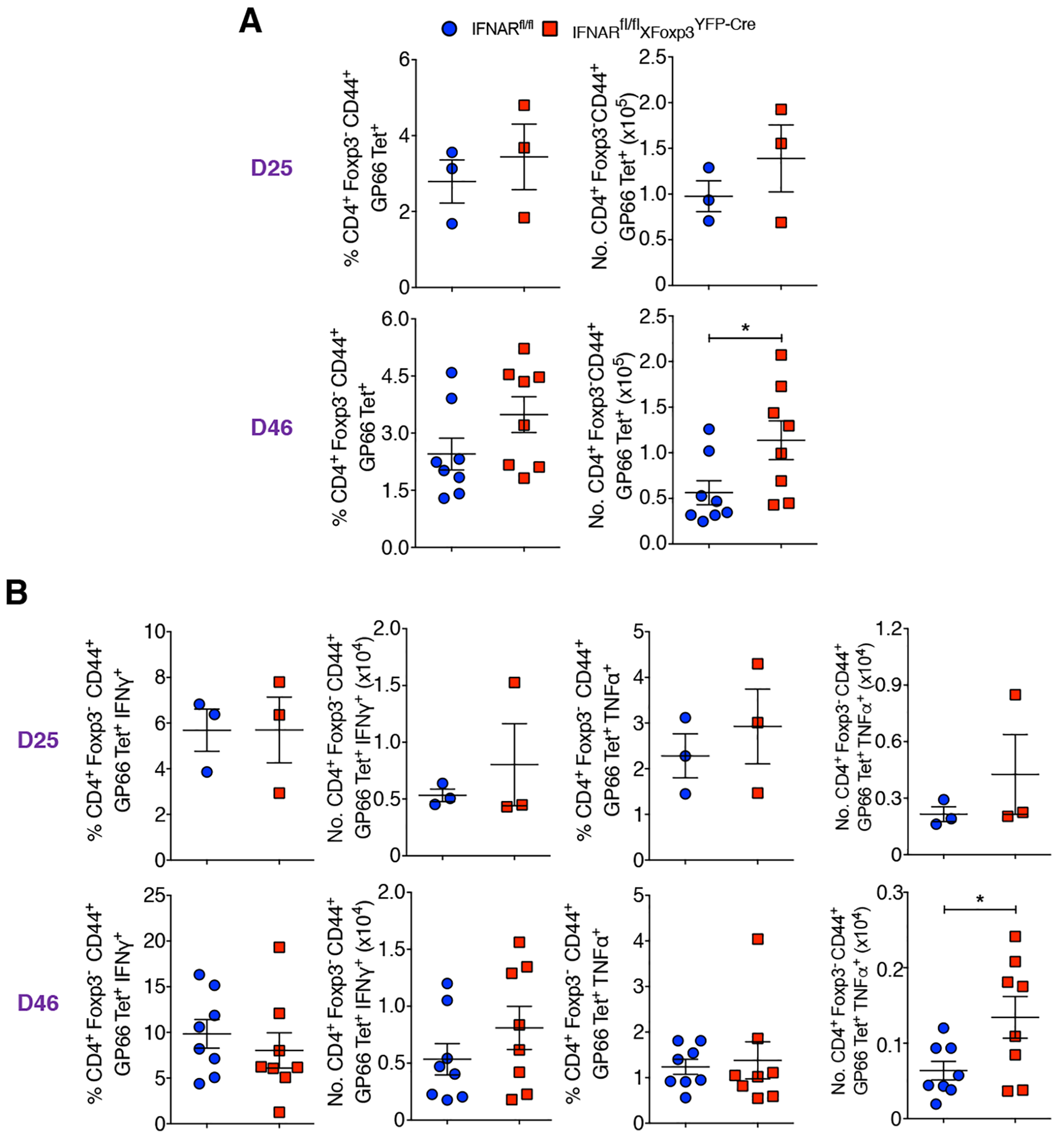

Supplement: S3 Fig — (A) Spleen cells from chronic LCMV infected (day 25 and day 46) IFNARfl/fl and IFNARfl/fl x Foxp3YFP-Cre mice were analyzed for GP66 Tet+ within CD4+Foxp3-CD44+ T cells. (B) Spleen cells from day 25 and day 46 Cl-13 infected mice were stimulated with GP61. Frequencies and absolute numbers of IFN-γ+ and TNF-α+ cytokine producing cells within CD4+Foxp3-CD44+GP66 Tet+ T cells are shown. * P < 0.05 (unpaired two-tailed Student’s t-test). Data are shown from one to two representative experiments (A and B) involving three to eight mice per group (Mean±SEM). (TIF) [file ppat.1006985.s003.tif]

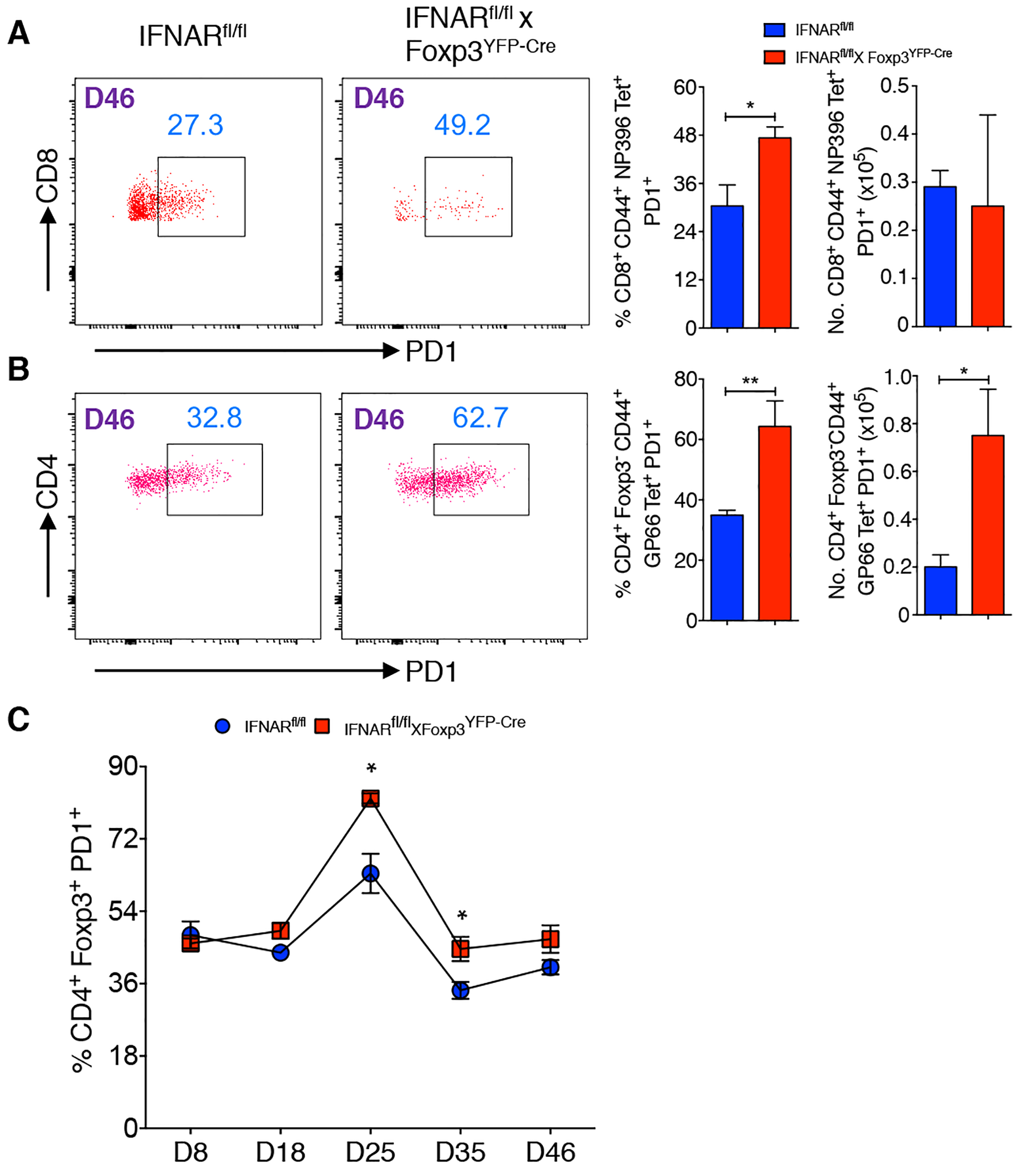

Supplement: S4 Fig — PD1 expression was evaluated within CD8+CD44+NP396 Tet+ T cells (A), and CD4+Foxp3-CD44+GP66 Tet+ T cells (B) from day 46 Cl-13 infected mice. (C) Kinetics of PD1 expression is shown on gated CD4+Foxp3+ T cells during chronic LCMV infection. * P < 0.05 and ** P < 0.01 (unpaired two-tailed Student’s t-test). Data are shown from a representative of two experiments (A and B), and from five experiments (C) with three to four mice per group in each experiment (Mean±SEM). (TIF) [file ppat.1006985.s004.tif]

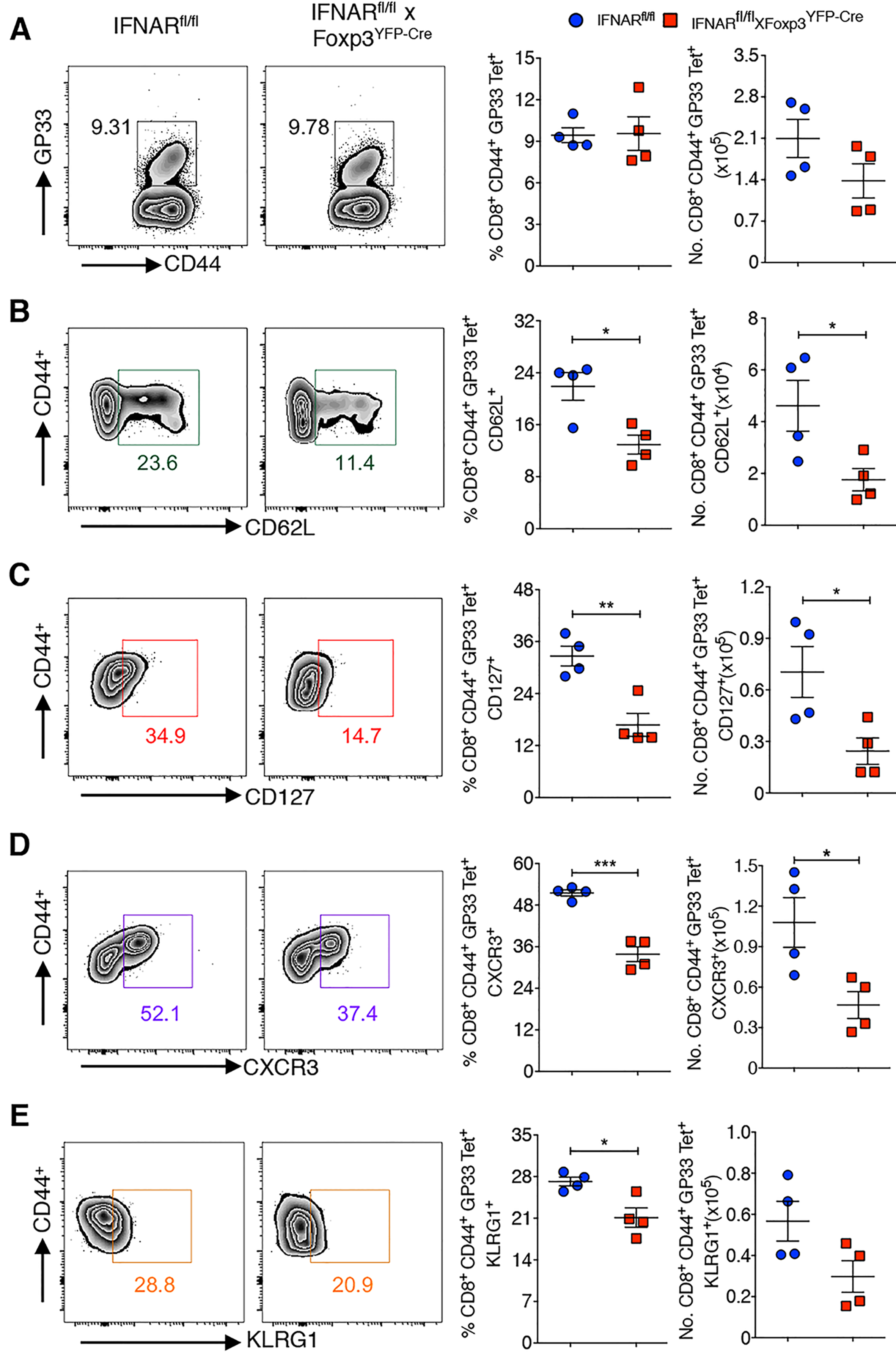

Supplement: S5 Fig — (A) Spleen cells from chronic LCMV infected (day 46) IFNARfl/fl and IFNARfl/fl x Foxp3YFP-Cre mice were analyzed for frequencies and absolute numbers of GP33 Tet+ T cells. Frequencies and absolute numbers of CD62L+ (B), CD127+ (C), CXCR3+ (D), and KLRG1+ (E) cells were determined within gated CD8+CD44+GP33 Tet+ T cells as in A. * P < 0.05, ** P < 0.01, and *** P < 0.001 (unpaired two-tailed Student’s t-test). Data obtained from a representative of two experiments (A-E) involving four mice per group in each experiment (Mean± SEM). (TIF) [file ppat.1006985.s005.tif]

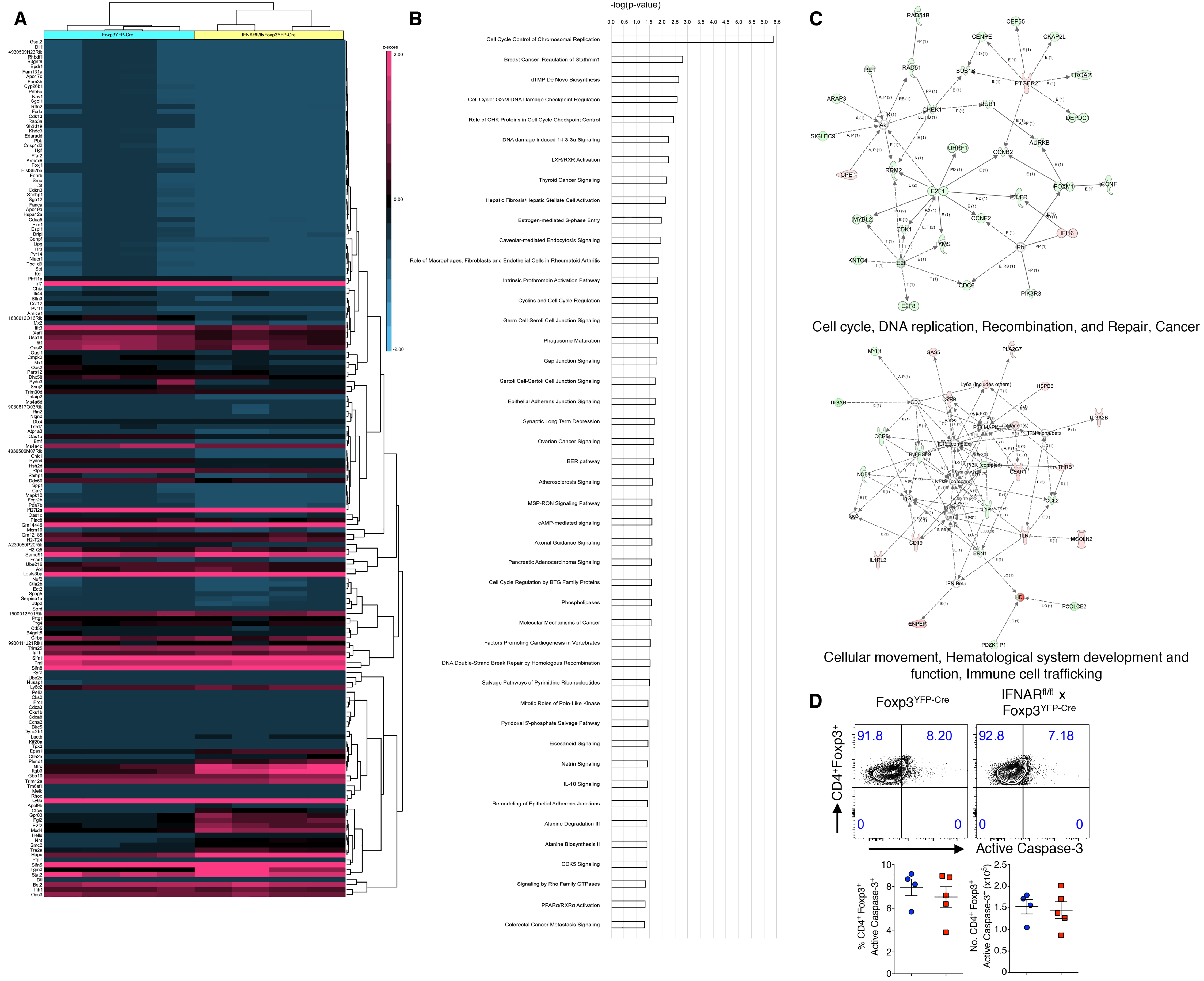

Supplement: S6 Fig — (A) Day 5 LCMV Armstrong infected Foxp3YFP-Cre and IFNARfl/fl x Foxp3YFP-Cre mice sorted CD4+YFP+ Treg cells were analyzed through RNA-seq (4 samples in each group), heat map showing the significant differential expression of 174 IFN-related genes, differentially expressed genes were normalized by z-score (fold change 1.5 and above, adjusted P < 0.05). (B) Top canonical pathways derived from IPA of differentially expressed non-IFN related genes from Tregs of Foxp3YFP-Cre and IFNARfl/flxFoxp3YFP-Cre mice during day 5 LCMV Armstrong infection were shown (adjusted p value < 0.1). (C) Top two networks were obtained by IPA based on co-expression, transcription factor binding site predictions and protein-protein interactions (genes in green are downregulated, whereas red are upregulated in Foxp3YFP-Cre mice). (D) Frequencies and total numbers of CD4+Foxp3+ Tregs positive for Active Casapse-3 cells are shown from day 5 acute LCMV infected mice. Transcriptome data obtained from an experiment involving four mice per group (A-C), and Active caspase-3 detection involved an experiment with four to five mice per group (D). (TIF) [file ppat.1006985.s006.tif]

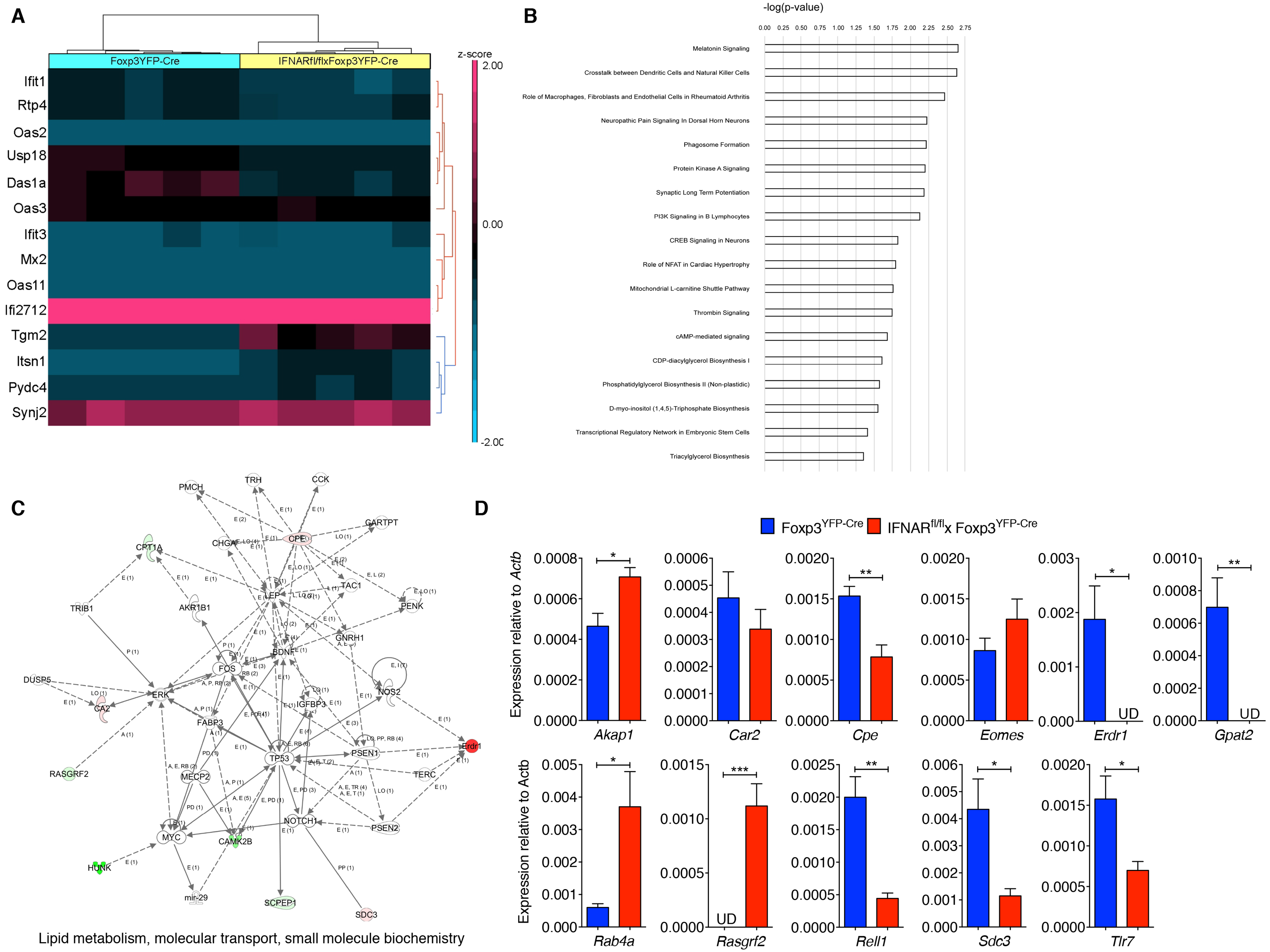

Supplement: S7 Fig — (A) Day 25 LCMV Cl-13 infected Foxp3YFP-Cre and IFNARfl/fl x Foxp3YFP-Cre mice sorted CD4+YFP+ Treg cells were analyzed through RNA-seq (5 samples in each group), heat map showing the significant differential expression of 14 IFN-related genes, differentially expressed genes were normalized by z-score (fold change 1.5 and above, adjusted P < 0.05). (B) Top canonical pathways obtained from IPA of differentially expressed non-IFN related genes from Tregs of Foxp3YFP-Cre and IFNARfl/flxFoxp3YFP-Cre mice during day 25 LCMV Cl-13 infection were shown (adjusted p value < 0.1). (C) Top network is derived by IPA based on co-expression, transcription factor binding site predictions and protein-protein interactions (genes in green are downregulated, whereas red are upregulated in Foxp3YFP-Cre mice). (D) Sorted CD4+YFP+ T cells cDNA samples from LCMV Cl-13 (post day 25) infected Foxp3YFP-Cre and IFNARfl/fl x Foxp3YFP-Cre mice were subjected to qPCR analysis. Gene expressions of Akap1, Car2, Cpe, Eomes, Erdr1, Gpat2, Rab4a, Rasgrf2, Rell1, Sdc3, and Tlr7 were calculated in relative to the Actb expression. * P < 0.05, ** P < 0.01 and *** P < 0.001 (unpaired two-tailed Student’s t-test). U.D., undetectable levels. Transcriptome data obtained from an experiment involving five mice per group (A-C), and data is a representative of an experiment involving five samples obtained from five mice per group (D) (Mean±SEM). (TIF) [file ppat.1006985.s007.tif]

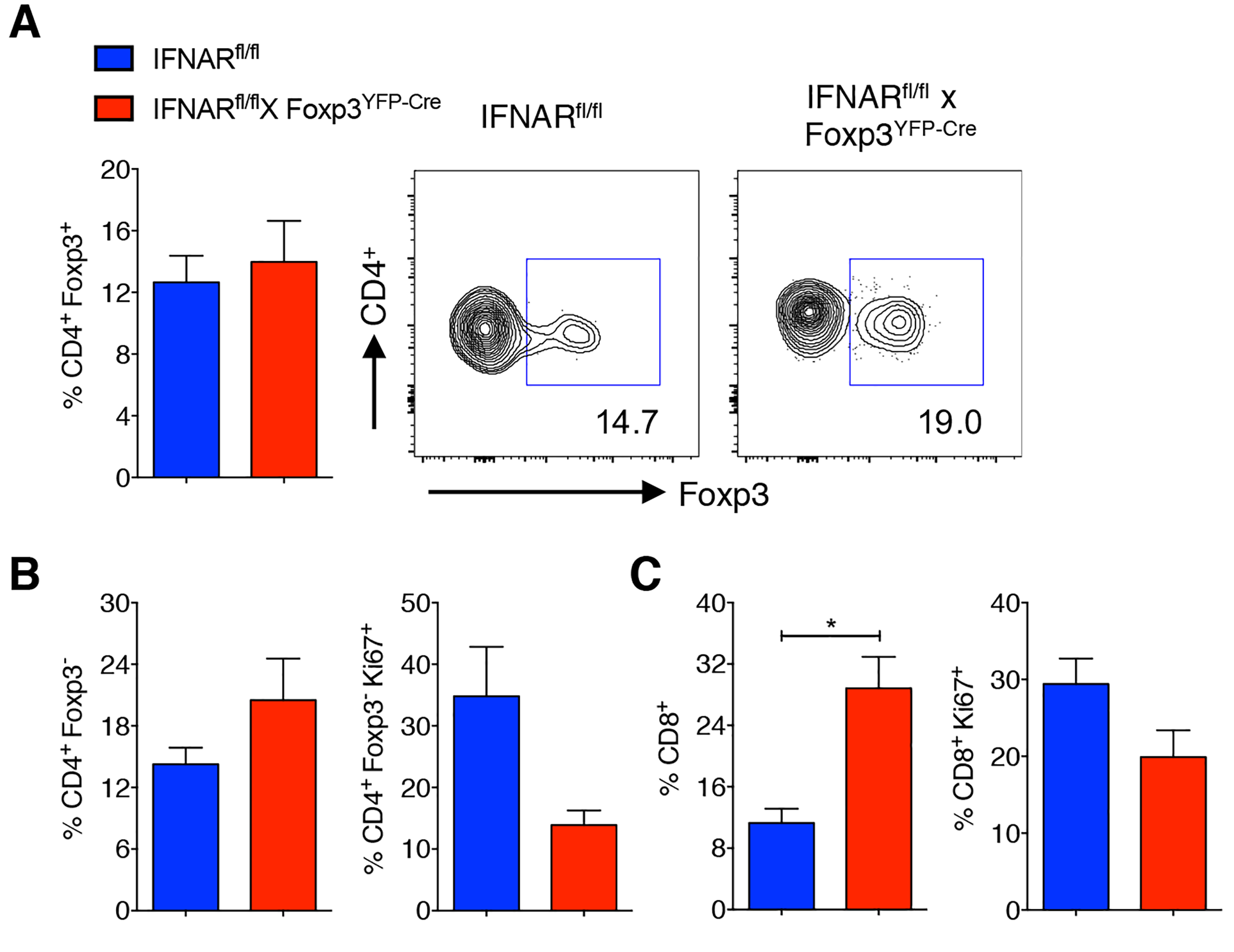

Supplement: S8 Fig — (A) IFNARfl/fl and IFNARfl/fl x Foxp3YFP-Cre mice were injected with 2x10^5^ MC38 cells subcutaneously. On day 18 post tumor implantation TIL were isolated, CD4+Foxp3+ T cell frequencies were ascertained. (B and C) Ki67 expression by tumor infiltrating CD4+Foxp3- and CD8+ T cells was evaluated. * P < 0.05 (unpaired two-tailed Student’s t-test). Data shown from two experiments (A), and from a representative of two independent experiments (B and C) involving three to five mice per group in each experiment (Mean±SEM). (TIF) [file ppat.1006985.s008.tif]
